# Theoretical Investigation of Charge Modulation Effects in Two Pyridine-Based Fluorescence Probes for Nerve Agent and Acetylcholinesterase (AChE) Detection

**DOI:** 10.1021/acsomega.5c06101

**Published:** 2025-10-03

**Authors:** Matheus Máximo-Canadas, Bruno Gabriel Motta Rodrigues, Itamar Borges

**Affiliations:** Departamento de Química, 28098 Instituto Militar de Engenharia (IME), Praça General Tibúrcio, 80, Rio de Janeiro, RJ 22290-270, Brasil

## Abstract

The efficient detection of nerve agents is paramount in civilian and war contexts. In this work, we investigated theoretically the mechanisms of fluorescence quenching and charge transfer (CT) in two recently synthesized small molecule fluorescent probes for detecting nerve agents, NMU-1 and 10-hydroxybenzo­[*h*]­quinoline (HBQ-AE), which are based on the pyridine group as the identifying unit. These fluorescent molecules change their emission pattern upon binding to an organophosphorus compound. We employed density functional theory (DFT), implicit methods for describing the water solvent, and a path integral approach to calculate fluorescence rates and emission spectra from first-principles for analyzing the interaction between the nerve agent simulant diethyl chlorophosphite (DCP) and NMU-1 or HBQ-AE. Upon interacting with DCP, both HBQ-AE and NMU-1 experience strong fluorescence quenching, with intensity reductions of ∼99.65 and ∼99.95%, respectively, due to enhanced CT induced by DCP’s electron-accepting nature and formation of a positively charged nitrogen within the probes. Although the measured fluorescence enhancement in HBQ-AE/DCP systems was attributed to the HBQ-DCP product, our results show a different picture: the true fluorescent species is the hydrolysis product HBQ + H, whose calculated emission differs by a blue shift of only 0.23 eV from the experimental data, rather than the HBQ-DCP product as previously thought. For acetylcholinesterase (AChE) detection, fluorescence is confirmed to originate from the HBQ-Keto, with a blue shift deviation of 0.12 eV. The NMU-1 calculations also confirm the experiments, showing a 0.24 eV deviation, thus confirming its quenching-based detection of DCP. Therefore, HBQ-AE is a fluorescence turn-on sensor via its emissive hydrolysis product HBQ+H. In contrast, NMU-1 is a turn-off sensor, as both NMU-DCP and NMU+H are essentially nonemissive. Our theoretical approach is general, accurate, and can be applied to different problems involving fluorescent probes and binding agents.

## Graphical abstract

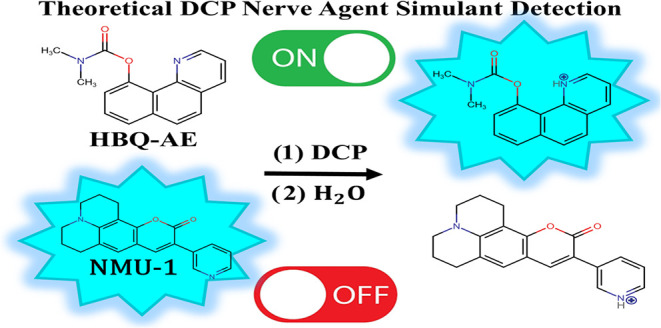

## Introduction

Fluorescence-based detection is pivotal in analytical chemistry and biomedical diagnostics due to its remarkable sensitivity, selectivity, and real-time detection capability.^1–3^ In most cases, they are based on fluorescence probes, molecules that modify their fluorescence emission when bonded to specific types of molecules. This property enables the detection of a target after it has selectively attached to a specific area or functional group of the molecule, as the original fluorescence signal changes.^4^

The fluorescent probes typically consist of two components: a selective receptor capable of interacting with the analyte and fluorophore units that convert molecular and environmental information into a measurable fluorescence signal by emitting distinct fluorescence or by shifting, enhancing, or quenching the fluorescence emission.^4,5^ These processes may be particularly influenced by charge transfer (CT) effects, as the probe-target assembly may function as a push–pull molecule or complex.^6^ CT is a common phenomenon in chemistry,^7^ materials science,^8,9^ biology, and medicine,^10^ and has several applications in optoelectronics,^11–13^ photovoltaics, and chemical sensors.^14–19^ Concerning fluorescence phenomena, although it is a widely well-known phenomenon, new insights are still being discovered.^20^

The interaction between the probe and analyte can change the fluorescence wavelength and intensity, enabling the detection of environmental molecules and biomolecules with remarkable sensitivity, often surpassing one part per trillion.^4^ The versatility of fluorescent probes stems from their simple synthesis, low cost, high sensitivity, rapid response, *in situ* and real-time detection capabilities, and strong temporal and spatial resolution.^5^ Due to these properties, fluorescent probes have been extensively used in many areas, including in chemical defense for detecting nerve agents (NAs).^21–24^ There are alternatives for detection, such as those based on mass spectrometry methods.^25–27^ NAs are highly toxic chemical warfare agents that can be absorbed through skin, inhalation, and ingestion.^28^ They have become a significant concern following events such as the assassinations in Malaysia (2017), Salisbury (2018), and Russia (2020), along with the continuing chemical weapons crisis in Syria since 2013.^29–31^ These incidents have underscored the threat posed by these agents and the urgent need for reliable and convenient detection methods.^32^ A NA primarily acts as an acetylcholinesterase inhibitor (AChE), disrupting signal transmission between nerves and muscles. This disruption can lead to a range of effects, from convulsions and paralysis to death, depending on the level of exposure and dosage.^33,34^

Tabun (GA), Sarin (GB), and Soman (GD) are common NAs.^35^ They are colorless liquids, soluble in water and organic solvents, volatile, and bear a fundamental organophosphorus structure characterized by a phosphorus atom bonded to oxygen in the form of an ester, as shown in Scheme 1. For experimental investigations, diethyl chlorophosphite (DCP) is a widely employed simulant because it reduces the risk associated with the high volatility of the NAs. Therefore, fluorescent probes synthesized for the detection of NAs are frequently tested with the DCP molecule.^36–39^ The DCP molecule itself has also been the subject of theoretical investigations.^40–42^

**Scheme 1:**
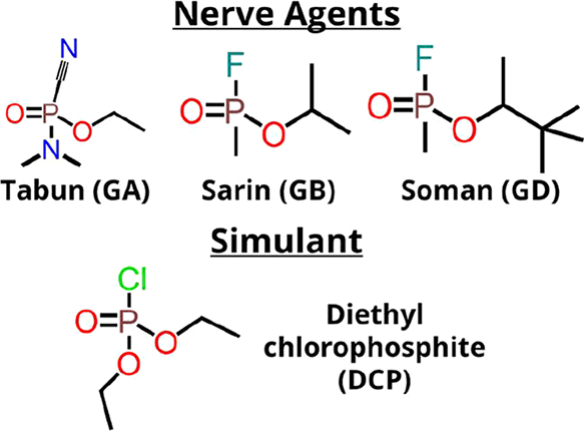
Tabun, Sarin, and Soman Nerve Agents and Their Simulant Diethyl Chlorophosphite (DCP)

Despite significant progress in synthesizing fluorescent probes, the detailed electronic mechanisms governing their response to organophosphorus compounds remain insufficiently understood. This knowledge gap hinders the rational design of more selective and efficient sensing systems.

In this work, we investigated theoretically using density functional theory (DFT) two recently synthesized fluorescent probes based on the pyridine group as the identifying unit, named NMU-1,^37^ and HBQ-AE.^36^ These molecules, when deposited on a test paper, undergo a color change upon binding to an organophosphorus compound. We elucidate and rationalize the electronic processes that occur when an organophosphorus substance binds to these probes and AChE. We used the DCP, a simulant of the NA Tabun, for the simulations.

## Theoretical and Computational Methods

We employed the B3LYP^43,44^ DFT hybrid functional and the def2-TZVP­(-f)^45^ Karlsruhe basis set for the optimization and frequency calculations of the singlet ground state (*S*_0_) and the first singlet excited electronic state (*S*_1_). To accelerate the calculation of the two-electron integrals, the resolution of identity approximation was applied to the Coulomb term (RIJ), while the exchange term was handled using the chain of spheres algorithm (COSX), along with the appropriate auxiliary basis set (def2/J and def2-TZVP/C).^46,47^ The TightSCF convergence criterion was applied. The solvation was implemented using the implicit Conductor-like Polarizable Continuum Model (CPCM)^48,49^ with water as the solvent. The Orca package version 6.0.1 was used for all electronic calculations and geometry optimizations.^50^

Section 1 of the Supporting Information (SI) collects the Cartesian coordinates of the optimized geometries and the charge transfer (CT) decompositions (see below), and Section 2 presents test calculations on NMU-1 and HBQ+H, which show that inclusion of D3 dispersion corrections has a small effect on optimized geometries. Section 2 also presents CT decomposition using the CAM-B3LYP^51^ range-separated functional, known to improve the description of CT excitations.^8,9,52^ The CAM-B3LYP results confirm that the overall pattern of CT contributions is maintained with respect to the B3LYP results. Therefore, all the subsequent analyses in this work are performed at the B3LYP/def2-TZVP­(-f)/CPCM­(water) level.

To overcome convergence challenges observed during the conventional geometry optimization of the HBQ-AE and HBQ-DCP systems, we employed the Global Optimization and Thermodynamics (GOAT)^53^ algorithm implemented in Orca. GOAT is a stochastic search protocol inspired by basin-hopping and minima-hopping strategies, designed to systematically explore the potential energy surface (PES) by iteratively performing random uphill displacements followed by local optimizations. This approach enables the identification of both the global minimum and low-energy conformational ensemble. In our calculations, GOAT was combined with the GFN2-xTB method to ensure computational efficiency while preserving the relevant potential energy surface (PES) features.^54^

The computation of the fluorescence emission employed a method based on the path integral approach,^55,56^ which uses the analytical resolution of Fermi’s Golden Rule-like equation from quantum electrodynamics and the Fourier transform of the Dirac delta function.^57^ This method is implemented in the Excited State Dynamics (ESD) module^55^ of the Orca software version 6.0.1.^50^

Mathematically, the fluorescence rate *k*(ω), at a photon angular frequency ω (=2πν), corresponding to the emission from an initial state *i* to a final state *f*, can be expressed in atomic units by eq 1:

1 $$k{\left(\omega \right)}_{\mathrm{if}}=\frac{2\omega^{3}}{3\pi c^{3}}\sum_{\mathrm{if}}P_{i}\left(T\right)\left\langle \Theta_{i}\left|\mathrm{\mu ̂}\right|{\mathrm{\Theta ̅}}_{f}\right\rangle \left\langle {\mathrm{\Theta ̅}}_{f}\left|\mathrm{\mu ̂}\right|\Theta_{i}\right\rangle \int e^{i\left(E_{i}-E_{f}-\omega \right)t}dt$$

Here, *c* denotes the speed of light in vacuum (∼2.9979 × 10^10^ cm·s^–1^); the |Θ_*i*_⟩ and |Θ̅_*f*_⟩ are the vibronic eigenfunctions of the *S*_0_ and *S*_1_ states, respectively, associated with vibrational levels *i* and *f*. The terms *E*_*i*_ and *E*_*f*_ refer to their total vibronic energies (electronic plus vibrational). The operator μ̂ is the electric dipole moment. The time integral represents the Fourier transform of the Dirac delta function, which enforces energy conservation. *P*_*i*_(*T*) is the Boltzmann population of vibrational level *i*, given by eq 2:

2 $$P_{i}\left(T\right)=\frac{e^{-\epsilon_{i}/k_{B}T}}{Z},,Z=\sum_{i}\exp\left[\frac{\epsilon_{i}}{k_{B}T}\right]$$

where ϵ_*i*_ is the vibrational energy of level *i* relative to the *S*_0_ zero-point energy, *k*_B_ is the Boltzmann constant (∼3.1668 × 10^–6^)­E_h_·K^–1^ (in atomic units), *T* is the absolute temperature (here 298.15 *K*), and *Z* the vibrational partition function. The rate eq 1 is solved by the path integral method.

The main advantage of this approach is its high theoretical accuracy, naturally incorporating vibronic couplings such as Herzberg–Teller (HT).^55^ In this framework, the electronic part of the transition dipole moment is assumed to vary with nuclear displacements, and then the matrix elements of μ̂ relative to the nuclear displacement can be expanded as shown in eq 3:

3 $$\mathrm{\mu ̂}\left(Q\right)={\mathrm{\mu ̂}}_{0}+\sum {\frac{\partial \mathrm{\mu ̂}}{\partial Q_{i}}|}_{Q=0}Q_{i}+...$$

where the *Q*_*i*_ is the dimensionless normal-mode coordinate for vibrational mode *i*, and μ̂_0_ = μ̂(*Q* = 0) corresponds to the zero-order (Franck–Condon, FC) term, i.e., the transition dipole moment taken at the equilibrium (FC) geometry, which neglects its variation with nuclear motion. Therefore, μ̂_0_ is the Franck–Condon term (coordinate-independent). The first-order derivative term gives the Herzberg–Teller contribution to the vibronic transition moment. The remaining terms represent higher-order terms, which are usually neglected within the HT approximation.

In the present work, the fluorescence simulations were performed using the Adiabatic Hessian (AH) model for the potential energy surface, which involves both *S*_0_ and *S*_1_ state Hessians (i.e., second derivatives matrix of the electronic energy with respect to the nuclear Cartesian displacements) explicitly computed. Vibrational contributions were included via the Herzberg–Teller approximation. Duschinsky rotations were neglected, and the spectra were broadened using a Voigt line-shape function with a Lorentzian width of 50 cm^–1^ and a Gaussian width of 200 cm^–1^. All calculations were carried out at 298.15 K in the harmonic normal mode coordinate system. The fluorescence rate constants *k*(ω) (eq 1) were extracted directly from the ESD output. Solvent effects on the ESD module were included via the CPCM model, and computed emission rates were subsequently scaled by the square of the solvent refractive index according to the Strickler–Berg relation.^58^

The Natural Transition Orbitals (NTOs)^59^ were computed using the TheoDORE package,^60,61^ which provides a clearer visualization of the excitation properties compared to the canonical molecular orbitals. If multiple NTO pairs significantly contribute to a transition, a transition amplitude (λ) is calculated to reflect the contribution of a specific NTO pair to the overall electronic transition. This λ quantifies the weight of a particular NTOs pair in describing the electron density redistribution after the transition. Generally, only one or a few NTO pairs exhibit appreciable amplitudes λ_*i*_. Consequently, the NTO decomposition offers a concise representation of the electronic excitation process.^60^

The TheoDORE package was also employed for a detailed examination of the one-particle transition density matrix (1TDM). It enables the evaluation of CT properties involved in the fluorescence emission, which is a *S*_0_ ← *S*_1_ electronic transition. For a molecule with two or more distinct regions or fragments, the representation of the 1TDM elements for a transition (denoted as *D*_*rs*_^0*n*^) from the ground state *S*_0_ or from an excited state (in our case, from *S*_1_) to the *n*th state *S*_*n*_ is expressed by eq 4, where the *ε̂*_*rs*_ is the excitation operator involving the *r* and *s* orbitals,

4 $$D_{\mathrm{rs}}^{0n}=\left\langle 0\left|{\mathrm{\varepsilon ̂}}_{\mathrm{rs}}\right|n\right\rangle$$

The descriptor CT number (Ω_*AB*_^*n*^) for excitation is defined by summing up the contributions from the fragments *A* and *B*, as indicated in eq 5. The *S* matrix is the orbital overlap, and the summations are performed over the basis functions associated with atoms μ and ν:

5 $$\Omega_{\mathrm{AB}}^{n}=\frac{1}{2}\sum_{\mu \in A}\sum_{\nu \in B}\left[{\left(D^{0n}S\right)}_{\mu \nu }{\left(SD^{0n}\right)}_{\mu \nu }+D_{\mu \nu }^{0n}{\left(SD^{0n}S\right)}_{\mu \nu }\right]$$

If *A* ≠ *B*, i.e., for different fragments, Ω_*AB*_^*n*^ represents the weight of CT from region *A* to *B*. In contrast, when *A* = *B*, i.e., for the same fragment, Ω_*AA*_^*n*^ is the weight of locally excited (LE) transitions on *A*. The total charge transfer number, *q*(*CT*), for a system comprising multiple fragments or regions, is determined by summing the off-diagonal elements as shown in eq 3 below. The term Ω^*n*^ is the normalization factor, representing the overall sum of CT numbers for all *A* and *B* pairs. The CT descriptor indicates the cumulative contribution of configurations where the initial and final orbitals are situated on separate fragments.

6 $$q\left(\mathrm{CT}\right)=\frac{1}{\Omega^{n}}\sum_{A}\sum_{B\neq A}\Omega_{\mathrm{AB}}^{n}$$

A value of *q*(*CT*) = 1 indicates the complete charge separation, whereas *q*(*CT*) = 0 corresponds to a locally excited or Frenkel excitonic state.^62^

For a given electronic excitation, the ORCA package identifies the excitation amplitudes (occupied → virtual single-excitation contributions). TheoDORE reconstructs the 1TDM from these amplitudes and, via a singular-value decomposition, derives the corresponding NTO pairs. This process is typically described as a redistribution of transition density from fragment *A* to fragment *B* (*A*→*B*), forming an electron–hole pair. During fluorescence, however, a radiative transition occurs between the first excited singlet *S*_1_ state back to the *S*_0_ (ground) state. In this case, the CT is interpreted as occurring in the reverse direction (*A* ← *B*), which represents the recombination of the electron with the hole and the restoration of the ground-state electronic density.

We have computed Huang–Rhys (HR) factors for all systems. The HR factor, originally introduced by Huang and Rhys in 1950,^63^ is a dimensionless quantity that characterizes electron–phonon (vibronic) coupling. It has since been extensively employed to investigate a wide variety of material properties.^64^ These factors provide a direct link between the nature of the excited-state relaxation pathways and the observed (or absent) fluorescence intensities. In this work, the HR factors quantify the degree of vibronic coupling between the first excited singlet state (*S*_1_) and its vibrational modes. Large HR values indicate significant displacements between the minimum of the *S*_0_ and *S*_1_ potential energy surfaces, resulting in redistribution of emission intensity from the 0–0 transition into vibrational progressions. Such displacements are also directly related to the magnitude of the Stokes shift.^65,66^ Large HR values, particularly for low-frequency modes, are known to promote nonradiative deactivation, for example, through internal conversion (IC), thereby reducing fluorescence quantum yields. In practice, the HR factors are computed with the ESD module of the Orca package by setting the keyword PRINTLEVEL to 3 or higher; in the present work, we employed a value of 4.

## Results and Discussion

### The HBQ-AE Fluorescence Probe

The Cartesian coordinates of the HBQ-AE and NMU-1 optimized geometries of both the ground (*S*_0_) and excited (*S*_1_) states in aqueous solution are presented in Table S1. The *S*_0_ converged structures are depicted in Figures S1–S7. We begin by discussing the HBQ-AE probe.

To rationalize the molecular basis of the sensing mechanism of HBQ-AE, we examined its structural and electronic features in isolated form and after binding to the DCP simulant and AChE. Figure 1 presents the suggested detection reaction proposed by Meng *et al*., who synthesized the probe.^36^ It represents the detection of DCP by the HBQ-AE probe and shows the proposed AChE detection reaction. The phosphorus in the DCP molecule is *sp* ^3^ *d* hybridized and thus can form five bonds: a double bond with an oxygen atom and three single bonds with ethoxy groups and chlorine. This structural arrangement suggests that DCP acts as an electron-accepting agent. Oxygen, more electronegative than phosphorus, attracts electron density away from the phosphorus through the double bond, resulting in an electron-deficient phosphorus atom. Chlorine, also more electronegative than phosphorus, similarly attracts electron density. Although the two ethoxy groups can donate electron density to the phosphorus atom, this contribution is relatively minor compared to the electron-accepting effects of the other substituents. In the isolated solvated HBQ-AE molecule, the electron density is predominantly concentrated in the pyridine ring, as will be discussed. The nitrogen’s lone pair, which does not participate in the resonance, remains available for electron donation. Consequently, a nucleophilic substitution occurs, as the HBQ-AE molecule bonds to DCP, leading to the elimination of the negative chlorine (Cl^–^). Chlorine acts as an excellent leaving group due to its high electronegativity, which allows it to efficiently stabilize the negative charge generated during the reaction. Therefore, the detection reaction of DCP by HBQ-AE must involve a nucleophilic substitution reaction.

**Figure 1:**
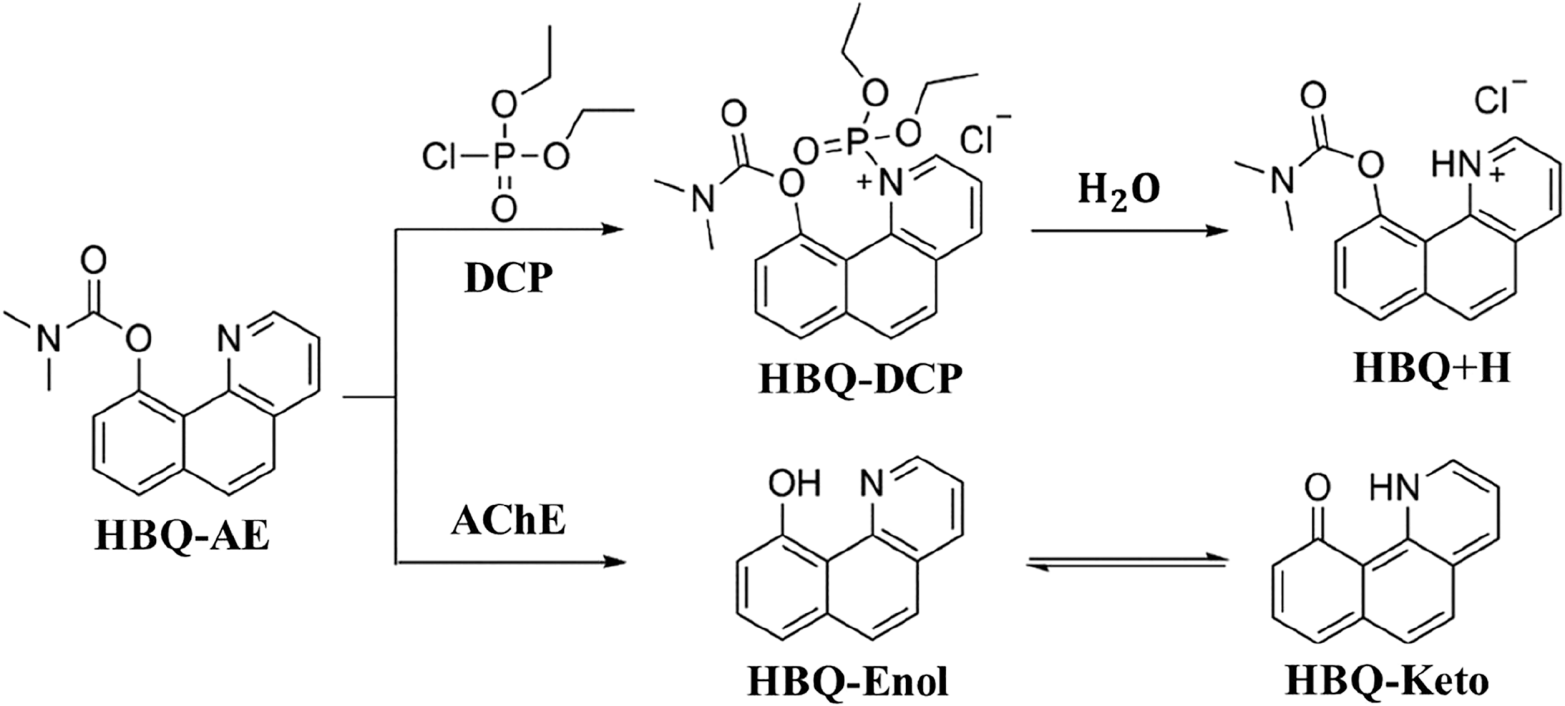
Proposed detection reaction of nerve agent simulant diethyl chlorophosphite (DCP) and acetylcholinesterase (AChE) by the fluorescent probe HBQ-AE. Adapted with permission from ref 36. Copyright 2020 Elsevier.

Figure 2a displays the computed fluorescence spectra at the B3LYP/def2-TZVP­(-f)/CPCM­(water) level of theory using the path integral-based method for the species involved in the reaction between the fluorophore HBQ-AE and the simulant DCP (probe-target) in aqueous solution. Notably, although HBQ-DCP exhibits a nonzero fluorescence signal, its emission peak is negligible compared to the other species in the reaction, about 99.65% less than the HBQ-AE intensity. This strongly suggests that the bonded DCP quenches HBQ-AE fluorescence.

**Figure 2:**
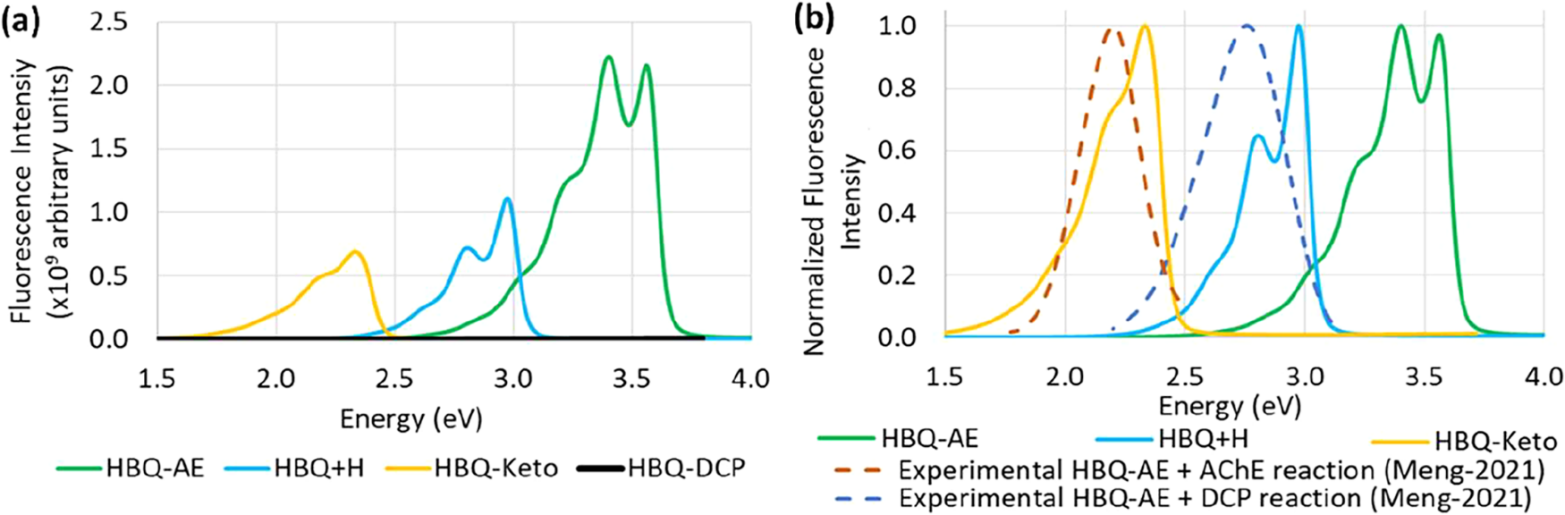
(a) Computed fluorescence spectra (intensity in arbitrary units) as a function of the energy in eV for the species HBQ-AE, HBQ-DCP, HBQ+H, and HBQ-Keto. (b) Normalized fluorescence intensities of the calculated spectra for HBQ-AE, HBQ+H, and HBQ-Keto systems. The experimental fluorescence spectra obtained from the reactions of the probe HBQ-AE with the targets DCP and AChE in aqueous solution are also displayed as dotted lines on (b).

Meng *et al.* reported HBQ-AE as a nonfluorescent molecule.^36^ However, Figure 2a clearly shows that HBQ-AE corresponds to the most intense fluorescence peak among all the species involved in the mechanism of Figure 1. The discrepancy arises from the spectral energy of the computed emission: the calculated maximum for HBQ-AE is 3.40 eV, which lies in the ultraviolet region, outside the visible range. Moreover, the experimental setup by Meng *et al.* employed a Hitachi F-2710 fluorescence spectrophotometer operating in the emission windows of ∼2.06–3.18, ∼1.91–3.10, and ∼ 1.77–2.48 eV, ranges that would not capture emission above ∼3.18 eV.^36^ Therefore, the computed intense UV fluorescence of HBQ-AE went undetected in their measurements, while the measured zero-emission region was reproduced.

The experimental group observed that adding DCP significantly enhanced the fluorescence intensity of the HBQ-AE solution, with higher concentrations of DCP correlating with a stronger emission around 2.75 eV.^36^ They attributed this emission to the formation of the HBQ-DCP product. However, our theoretical calculations place the emission maximum of HBQ-DCP at 3.79 eV, which differs substantially from the experimentally observed emission by 1.04 eV. Furthermore, as previously noted, HBQ-DCP’s emission is essentially negligible. In contrast, the HBQ+H molecule exhibits a calculated fluorescence peak at 2.98 eV, much closer to the experimental value, with a blue shift difference of only 0.23 eV. This result suggests that the fluorescence signal experimentally attributed to HBQ-DCP actually originates from the hydrolysis product HBQ+H. As shown in Figure 2b, the calculated peak for HBQ+H closely overlaps with the experimental peak. This interpretation is reinforced by the fact that the experimental probe HBQ-AE was used in an aqueous solution, where hydrolysis is expected to occur, as indicated by the reaction in Figure 1.

When the target is AChE instead of DCP, *Meng et al*. reported a fluorescence peak at 2.21 eV.^36^ We assign this emission to the HBQ-Keto species, for which our theoretical calculations predict a peak at 2.33 eV. This result has a slight blue shift of 0.12 eV compared to the observed value. Furthermore, as shown in Figure 2b, there is a clear partial overlap between the calculated emission of HBQ-Keto and the experimentally observed band, further supporting this assignment.

The NTOs related to the *S*_0_ ← *S*_1_ emission of the HBQ-AE-based systems, namely HBQ-AE, the HBQ-DCP product, HBQ+H, and HBQ-Keto, are presented in Figure 3. It can be noted that, for all systems, the electron–hole separation indicates both CT and LE characters, further discussed in the following paragraphs.

**Figure 3:**
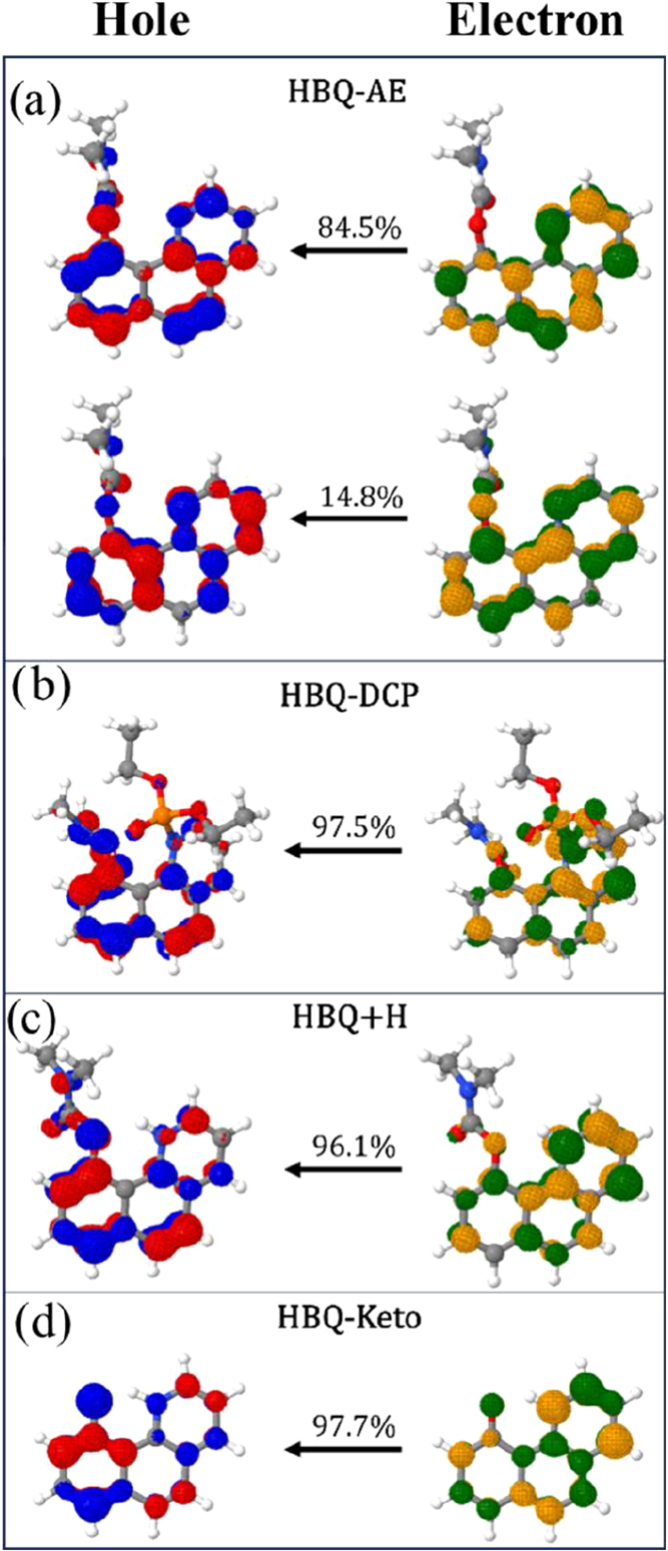
B3LYP/def2-TZVP­(-f)/CPCM­(water) Natural Transition Orbitals (NTOs) for the compounds of the HBQ group: (a) HBQ-AE, (b) HBQ-DCP product, (c) HBQ+H, and (d) HBQ-Keto, with the respective transition amplitude (λ).

To investigate charge transfer (CT) effects, the HBQ-AE molecule was partitioned into three fragments: *A*, *B*, and *C* as indicated in Table 1a. For HBQ-AE, the electron density is mainly localized at the benzo­[*h*]­quinoline moiety (fragments *B* and *C* together), with some CT from fragment *C* to fragment *B* having *q*_CT_(*CB*) = 0.356*e*, and a local excitation on the fragment *B* with *q*_LE_(*B*) = 0.338*e*. The lone pair on the nitrogen atom of the pyridine unit (fragment *C* in HBQ-AE) does not participate in the π-conjugation. This is due to the lone pair’s orientation within the molecular plane, perpendicular to the conjugated *p* orbitals, impeding its delocalization into the π system and contribution to aromatic resonance. Due to the high electronegativity of nitrogen, the pyridine ring predominantly acts as an electron acceptor. Consequently, upon photoexcitation within the HBQ-AE structure, an excited state with pronounced CT character from fragment *C* to fragment *B* is populated. Additionally, a very slight CT from fragments *B* and *C* to fragment *A* is observed (*q*_CT_(*BA*) = 0.019*e* and *q*_CT_(*CA*) = 0.016*e*). This behavior reflects the extended conjugation across the molecule. The nitrogen in fragment *A* has a lone pair that contributes to the π-conjugation through a positive mesomeric effect – unlike the nitrogen lone pair in fragment *C*. Moreover, the nitrogen in fragment *A* is bonded to two methyl groups, which enhances its electron-donating character. These findings are nicely visualized by the two NTO pairs in Figure 3a.

**Table 1:**
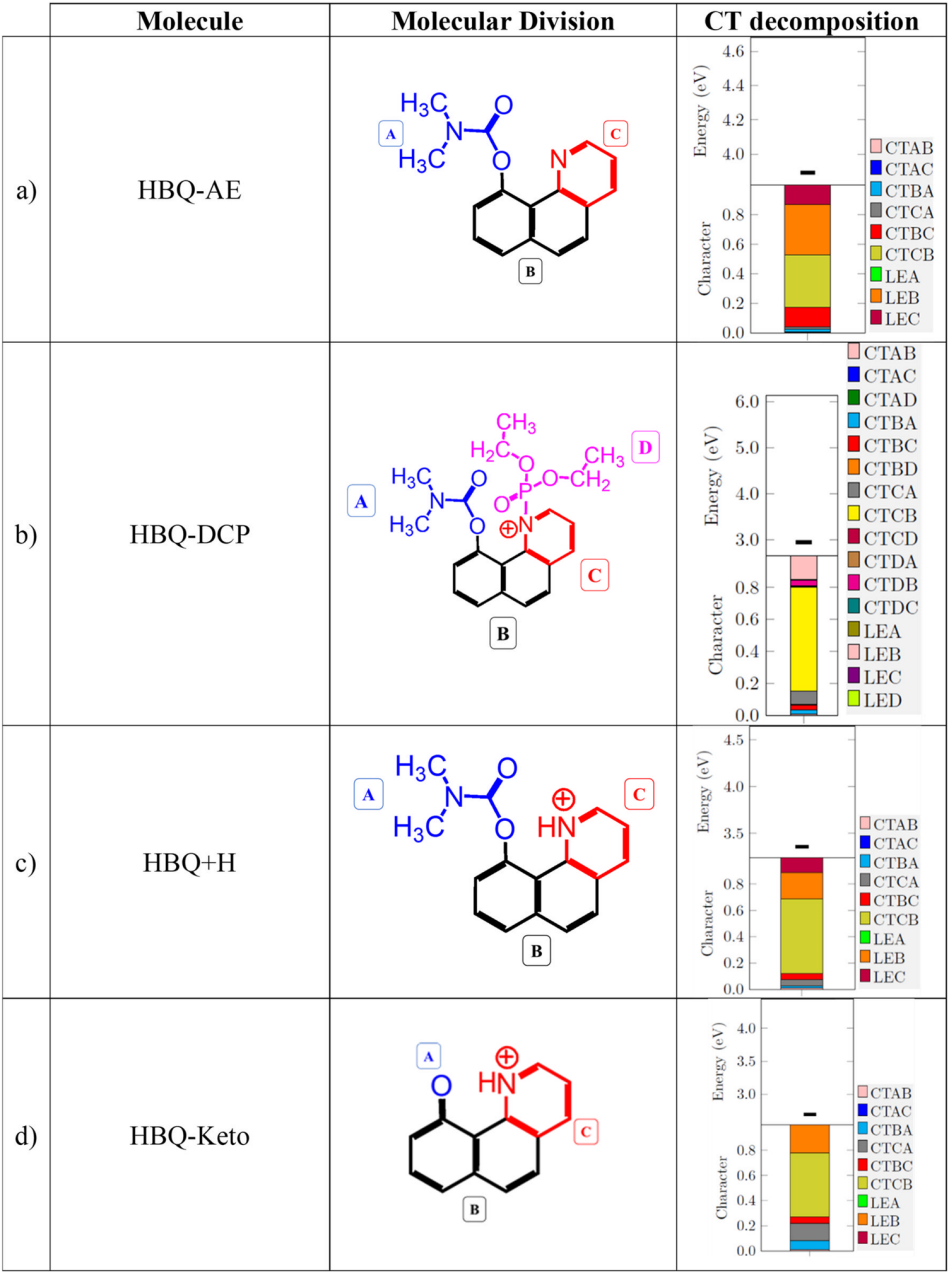
Molecular Structure of the Investigated Compounds of the HBQ Group: (a) HBQ-AE, (b) HBQ-DCP Product, (c) HBQ+H, and (d) HBQ-Keto, the Corresponding Charge Transfer (CT) Decomposition with the Respective Fluorescence Transition Energy and Contributions from Each Fragment^a^

a The molecular partitions (A–D) are highlighted. (CTXY: charge transfer from X → Y; LEX: local excitation on X).

The HBQ-DCP system was divided into four fragments for the CT analysis: *A*, *B*, *C*, and *D*—refer to Table 1b for identifying them. The explicit numerical values of the CT decomposition are presented in Table S2 of the SI. We found that the DCP simulant bonded to HBQ-AE induces a significant increase of the CT from fragment *C* to *B*, *q*_CT_(*CB*) = 0.356*e* for HBQ-AE to 0.649*e* for HBQ-DCP, an increase of approximately 82%. This is associated with a decrease of 75% of the *q*_CT_(*BC*) value, from 0.133*e* for HBQ-AE to 0.033*e* for HBQ-DCP. An increase of *q*_CT_(*CA*) from 0.016*e* to 0.083*e* is also observed. In contrast, the local excitation on fragment *B* is decreased, *q*_LE_(*B*) from 0.338*e* for HBQ-AE to 0.196*e* for HBQ-DCP, a 42% reduction. This behavior is due to the positively charged nitrogen in the fragment *C*, making it an even stronger electron acceptor compared to the HBQ-AE probe. Additionally, the high electronegativity of the DCP group influences an even more positive character of the nitrogen atom in HBQ-AE – it induces a greater CT to the ring, thereby intensifying CT from fragment *C*, which is adjacent to the DCP (labeled as fragment *D*), toward fragments *A* and *B*, due to the emission.

Regarding DCP (fragment *D*), upon emission, a slight CT primarily occurs from fragment *D* to fragment *B* (*q*_CT_(*DB*) = 0.035*e*). This is again due to the higher electronegativity of the DCP group. As fragment *C* also has an electron-acceptor character, there is a very small CT between fragments *C* and *D*, *q*_CT_(*DC*) = 0.006*e* and *q*_CT_(*CD*) = 0.005*e*. The NTOs in Figure 3b predominantly confirm a reduction of the electron density in fragments *C* and *D*, while showing an increase in fragments *A* and *B* due to the emission.

Upon hydration of the HBQ-DCP product, the DCP group is eliminated, and the pyridine ring (fragment *C*) becomes protonated, yielding the HBQ+H species. In this system, the CT more closely resembles that of HBQ-DCP than HBQ-AE, but with an intermediate CT character between them. For instance, the most pronounced effect is the CT from fragment *C* to *B* in all molecules, with *q*_CT_(*CB*) values of 0.356*e*, 0.569*e*, and 0.649*e* for HBQ-AE, HBQ+H, and HBQ-DCP, respectively. This scenario represents a 43% increase in CT from HBQ-AE to HBQ+H. The larger *q*_CT_(*CB*) value arises from the enhanced electron-accepting character of the protonated nitrogen in HBQ+H, relative to the neutral pyridine nitrogen in HBQ-AE. Consequently, due to emission, there is a larger CT from *C* to fragment *B*. However, the absence of the strongly electron-withdrawing DCP group in HBQ+H, compared to the HBQ-DCP system, results in a slightly attenuated CT effect relative to the probe-target product.

To sum up, protonation of the pyridine nitrogen in HBQ+H enhances the CT effect in HBQ-AE, although not to the same extent as in HBQ-DCP. In agreement with this analysis, the HBQ+H NTOs in Figure 3c reveal a decrease in the electron density of the fragment *C* and an increase in fragment *A*, compared to that hole of HBQ-AE (Figure 3a), thus indicating an enhanced CT process in HBQ+H.

Upon exposure to the AChE enzyme, the fragment *A* in HBQ-AE (see Table 1a) undergoes hydrolysis, forming a hydroxylated species, HBQ-Enol, which exists in resonance with its carbonyl counterpart, HBQ-Keto. Here, the analysis focuses on the HBQ-Keto form because it represents the thermodynamically favored structure due to its lower energy relative to HBQ-Enol.^27^ The CT pattern resembles that of HBQ-AE but shows an enhanced CT from fragment *C* to *B*. However, this enhancement is less pronounced than that observed for HBQ+H, wherein the nitrogen atom of fragment *C* is protonated, significantly amplifying its electron-withdrawing character. Quantitatively, *q*_CT_(*CB*) increases from 0.356*e* for HBQ-AE to 0.506*e* for HBQ-Keto, but to 0.569*e* for HBQ+H.

Now we discuss the Huang–Rhys (HR) factors. For HBQ-AE (Figure 4a), only one vibrational mode with a significant Huang–Rhys (HR) factor value was found, at a high frequency (1369 cm^–1^), related to the asymmetric stretching of the rings. Consequently, the calculated spectrum exhibits a non-negligible 0–0 transition, consistent with detectable fluorescence intensity. However, the lack of experimental emission may arise from the fact that the emission energy lies outside the visible range accessible to the spectrometer, as discussed above.

**Figure 4:**
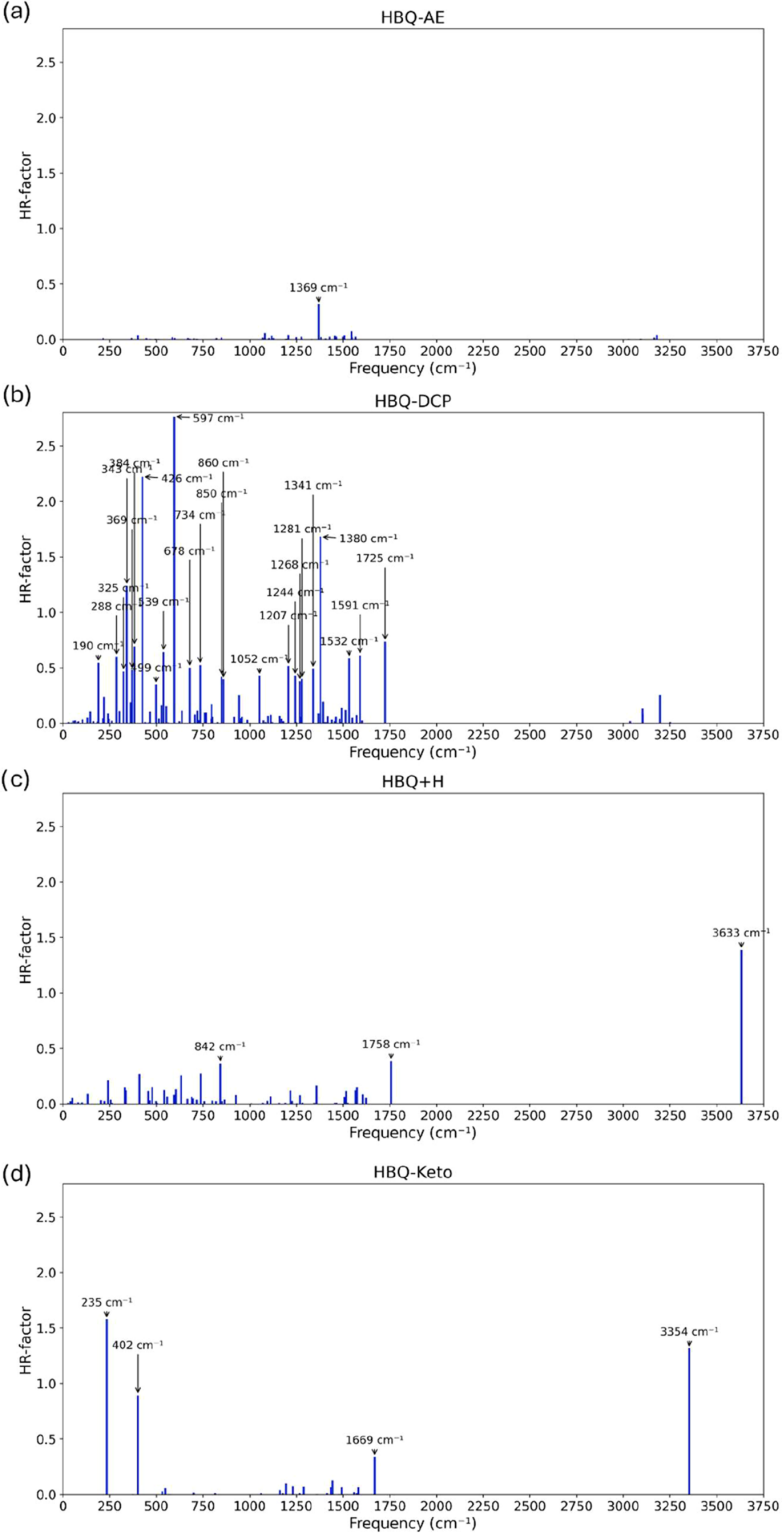
Calculated *S*_1_-*S*_0_ Huang–Rhys factors for the HBQ system, namely: (a) HBQ-AE, (b) HBQ-DCP, (c) HBQ+H, and (d) HBQ-Keto. The vibrational modes with large contributions to the Huang–Rhys factor are shown as insets.

In contrast, HBQ-DCP exhibits 24 vibrational modes with HR factors larger than 0.3, four of which exceed 1.0 (343, 426, 597, and 1380 cm^–1^)—see Figure 4b. The strong coupling of three low-to-medium frequency modes related to twisting provides efficient channels for vibronic coupling, promotes IC and effectively quenches the fluorescence, consistent with theoretical predictions.

After hydrolysis, HBQ+H displays only three modes with HR > 0.3 (842, 1758, and 3633 cm^–1^), with just the high-frequency mode 3633 cm^–1^ exceeding one (see Figure 4c), related to the asymmetric stretching of the rings, mainly involving fragment B. Although this mode contributes significantly to the HR factor, its high frequency limits its efficiency as a promoter of IC. As a result, HBQ+H is expected to retain a detectable emission, although weaker than HBQ-AE, in agreement with our theoretical spectrum and consistent with the experimentally observed fluorescence.

The AChE reaction product, HBQ-Keto, presents four modes with HR > 0.3, including two larger than 1 (235 and 3354 cm^–1^), as presented in Figure 4d. The low-frequency mode at 235 cm^–1^, related to the scissoring of fragments A and C, leads to substantial intensity reduction (≈38% lower compared to HBQ+H) and spectral broadening due to vibronic redistribution.

### NMU Fluorescence Probe

Figure 5 illustrates the proposed detection reaction of the DCP simulant by the NMU-1 fluorescent probe, as suggested by the group that synthesized it.^37^ This reaction closely resembles the detection pathway observed for the HBQ-AE probe depicted in Figure 1. For the NMU-1, the nitrogen lone pair on the pyridine moiety, which remains nonconjugated with the π-system, is readily available for a nucleophilic attack. Upon exposure to DCP, a nucleophilic substitution reaction occurs in which NMU-1 covalently binds to the target DCP, followed by the elimination of the chloride ion (*Cl* ^–^). Therefore, the detection of DCP by NMU-1 fundamentally proceeds through a nucleophilic substitution mechanism, similar to HBQ-AE. Importantly, both reactions do not terminate upon the initial formation of the NMU-DCP and HBQ-DCP products. Instead, in aqueous environments,^36,37^ the process can proceed further through a hydrolysis step. The water can then react with the probe-target product, eliminating the DCP target, followed by the protonation of the probe. Consequently, the NMU-DCP and HBQ-DCP products are converted into the protonated species NMU+H and HBQ+H, respectively. Figure 6a presents the computed fluorescence spectra of NMU-1, NMU-DCP product, and NMU+H, and Figure 6b compares the computed and experimental NMU-1 spectra.

**Figure 5:**
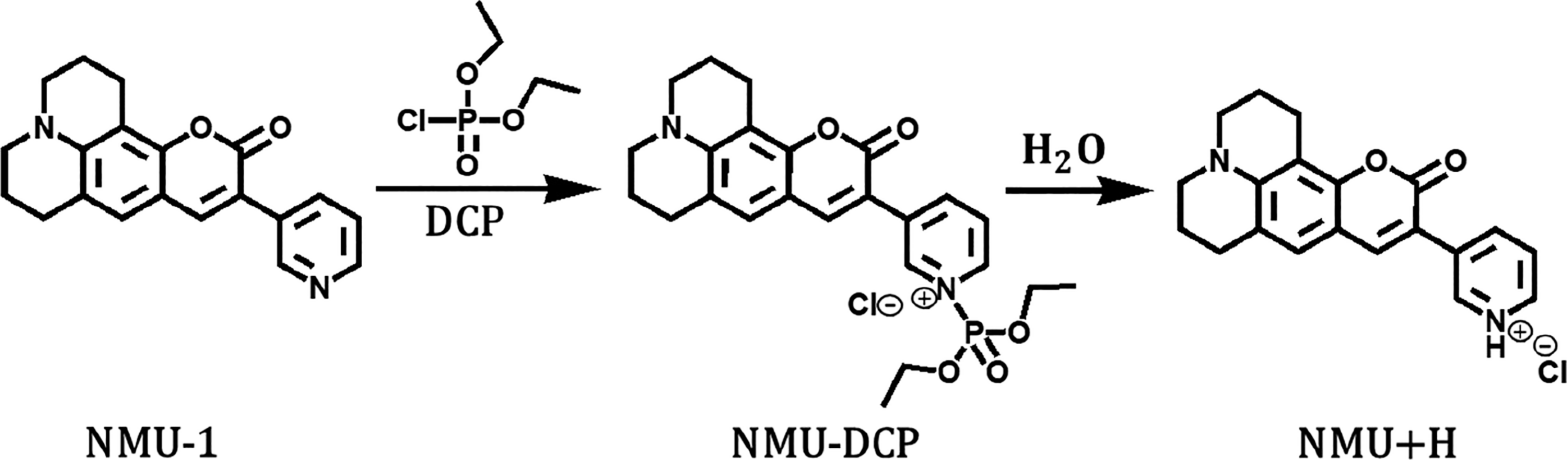
Proposed detection reaction of nerve agent simulant diethyl chlorophosphite (DCP) by the fluorescent probe NMU-1. Adapted with permission from ref 37. Copyright 2022 Elsevier.

**Figure 6:**
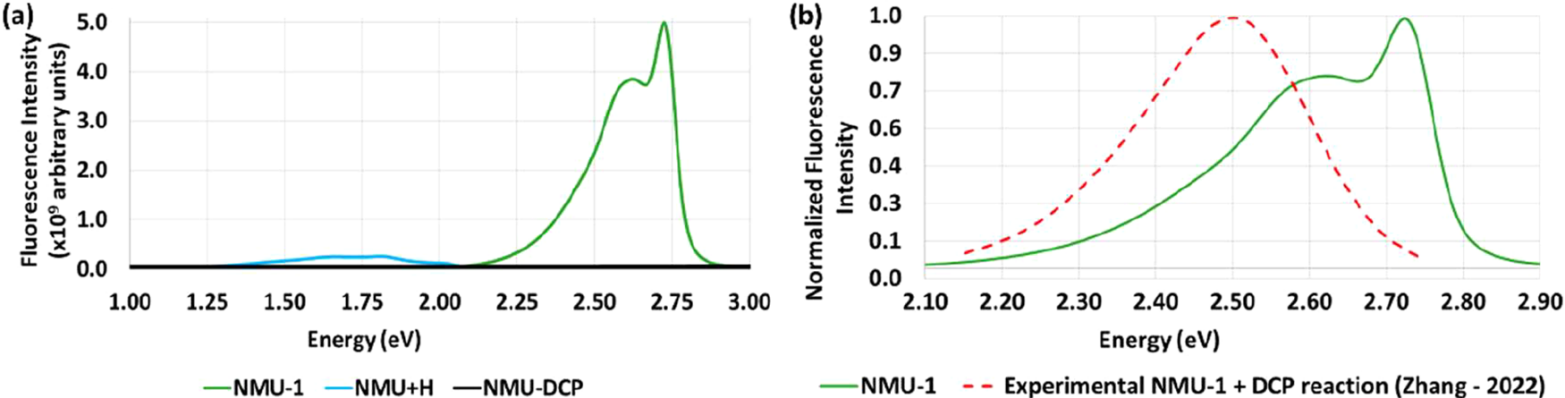
(a) Computed fluorescence spectra (intensity in arbitrary units) as a function of energy (eV) for the NMU-1, NMU-DCP, and NMU+H species. The experimental fluorescence spectra obtained from the reactions of the probe NMU-1 with the target DCP in aqueous solution are displayed as dotted lines on panel (b).

As shown in Figure 6a, the binding of the DCP target to NMU-1 results in significant fluorescence intensity quenching (about 99.95% of NMU-1), with the NMU-DCP (probe-target) emission being effectively absent from the experimental emission spectrum in Figure 6a. Thereby, the identification reaction of DCP with NMU-1 is through fluorescence quenching. Our result agrees with experimental observations, where increasing concentrations of DCP progressively decrease the fluorescence intensity until suppression.^37^ Upon hydrolysis, the NMU+H species is formed, which exhibits only a very weak, nearly negligible, computed fluorescence signal. This explains the near-complete fluorescence suppression observed by the experimental group. In contrast, the HBQ-AE system shows a distinct behavior: while the hydrolysis product HBQ+H still retains significant emission (corresponding to a reduction of only 49.93% compared to HBQ-AE), the NMU+H exhibits markedly lower fluorescence (with a decrease of 94.81% compared to NMU). As a result, HBQ-AE is a fluorescence turn-on sensor, capable of detecting DCP through the emission of its hydrolysis product (HBQ+H), rather than by the HBQ-DCP product as previously thought. In contrast, NMU-1 is classified as a fluorescence quenching probe (turn-off sensor), since both its hydrolysis product NMU+H and the NMU-DCP product are essentially nonemissive.

The experimental fluorescence peak of NMU-1 has been reported at approximately 2.49 eV.^37^ In comparison, our calculation predicts a maximum emission at 2.72 eV, corresponding to a difference of just 0.24 eV relative to the experimental value.

Similar to the 10-hydroxybenzo­[*h*]­quinoline (HBQ) based systems (HBQ-AE, HBQ-DCP, HBQ-keto, and HBQ+H), the *S*_0_ ← *S*_1_ transition in the NMU set (NMU-1, NMU-DCP, and NMU+H) also presents both CT and LE characters—see the electron–hole plots separation in Figure 7. For all members of the NMU systems, there is a high electron density localized on the pyridine unit. Following the emission, this density diminishes and becomes more pronounced on the coumarin-6H (C6H) unit. The C6H unit comprises the remainder of the molecule, excluding the pyridine ring – see the blue and black moiety for C6H and the red for the pyridine-based unit in the structures depicted in Table 2.

**Figure 7:**
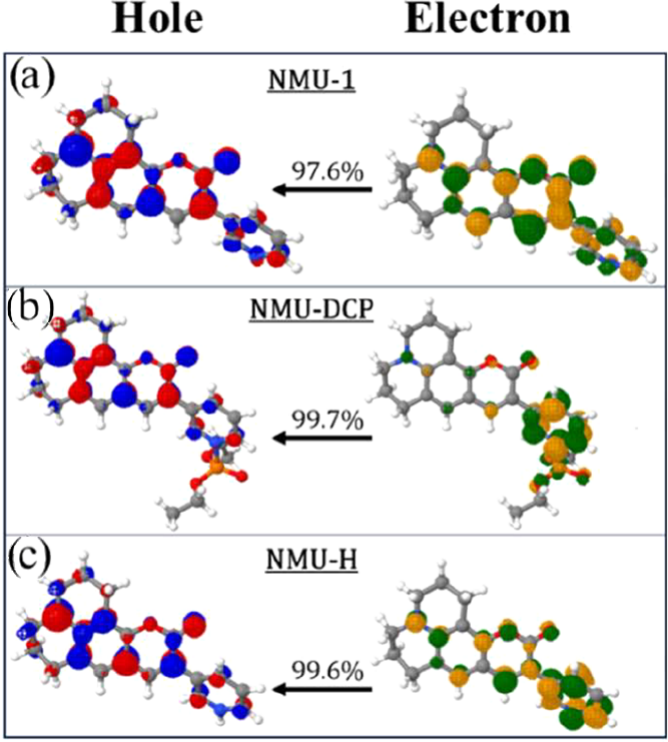
B3LYP/def2-TZVP­(-f)/CPCM­(water) Natural Transition Orbitals (NTOs) for the NMU derivatives with the respective transition amplitude (λ): (a) NMU-1, (b) NMU-DCP, and (c) NMU-H.

**Table 2:**
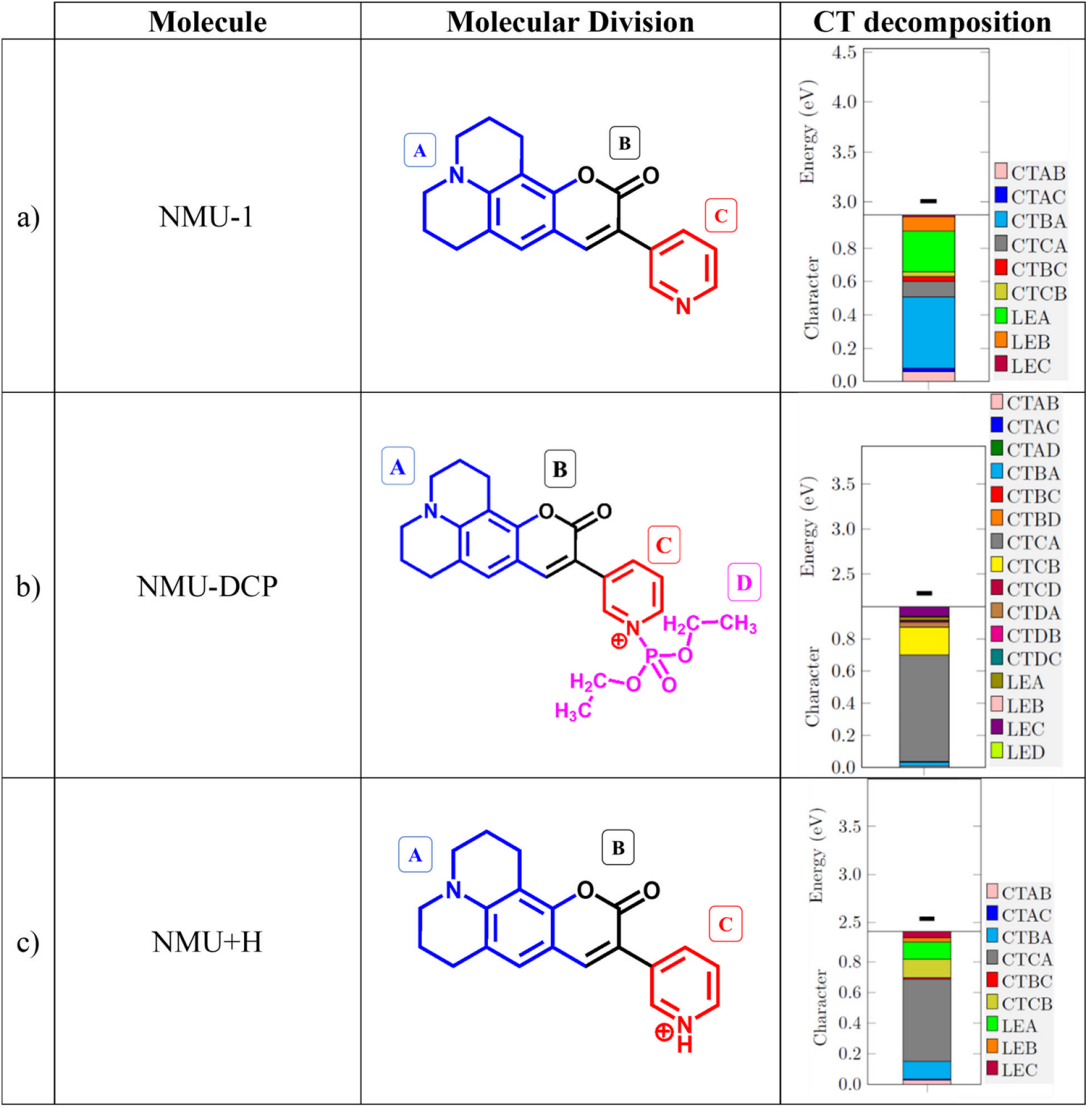
Molecular Structure of Each Compound of the NMU System: (a) NMU-1, (b) NMU-DCP, and (c) NMU+H, and the Computed Charge Transfer (CT) Values with the Respective Transition Energy^a^

a The molecular partitions (A–D) are highlighted.

Table 2 depicts the regions chosen for the CT analysis and their contributions. The numerical values of the CT decomposition are presented in Table S2 of the SI. We used the NTOs depicted in Figure 7 for this analysis. It was found some charge density in the julolidine-based unit, i.e., the tricyclic fused-ring system with nitrogen bridging the two outer rings, defined as fragment *A* in Table 2. The charge density on fragment *A* increases by the emission process, while for the ester group (fragment *B* in Table 2) and the pyridine ring (fragment *C* in Table 2), the charge density decreases. Moreover, the substitution reaction in the DCP group occurs through the nucleophilic center of the nitrogen in the pyridine ring. Consequently, the ring to which this nitrogen is attached was treated as a separate fragment (*C*).

The NMU-1 probe structure has two nitrogen atoms. The nitrogen in fragment *A* (the julolidine-based fragment in Table 2a) is *sp* ^*3*^ hybridized and has a lone pair in an orbital perpendicular to the plane of the ring, making them available for resonance with the rest of the molecule. In contrast, the nitrogen in fragment *C* (pyridine group) has a lone pair parallel to the plane of the ring, which does not participate in the resonance; thereby, this nitrogen atom contributes primarily through its intrinsic electronegativity. Consequently, fragment *A* is an electron-donating group within the molecule, while fragment *C* acts as an electron-accepting group. Additionally, the ester group (RCOOR), present in fragment *B* of NMU-1, is a stronger electron-acceptor than nitrogen. Our results confirm it: the highest CT value due to emission is from the electronegative ester group (fragment *B*) to fragment *A* (*q*_CT_(*BA*) = 0.426*e*). For the *S*_0_ → *S*_1_ excitation, electron density flows from the electron-donating fragment *A* to the electron-accepting fragment *B*, a CT process that is reversed upon emission (from *B* to *A*), as confirmed by the computed *q*_CT_(*BA*) value. The second most significant contribution corresponds to a LE in fragment *A* (*q*_LE_(*A*) = 0.245*e*), with the nitrogen lone pair delocalized throughout the ring. The third highest contribution for NMU-1 is a slight CT from fragment *C* to fragment *A* (*q*_CT_(*CA*) = 0.096*e*) due to the emission.

Similarly to the interaction of HBQ-AE with the DCP target, the NMU-1 molecule also has a nitrogen lone pair in fragment *C* that does not participate in resonance and is thus available for a nucleophilic attack. This can be a nucleophilic substitution of the nucleophile NMU-1 by the electrophile DCP, resulting in the elimination of a chloride anion (Cl^–^) and the formation of the NMU-DCP molecule (probe-target). In such a case, the ester group is no longer the predominant electron-accepting group, as it was on NMU-1. Instead, the nitrogen in fragment *C*, which was already an electron-accepting group, becomes positively charged, further enhancing its electron-accepting character. Additionally, this nitrogen atom is bonded to the electronegative DCP unit (fragment *D*). Consequently, for the NMU-DCP molecule, the major CT contribution during fluorescence is from fragment *C* to fragment *A* (*q*_CT_(*CA*) = 0.666*e*), distinctively from NMU-1, in which CT was from fragment *B* to fragment *A* (*q*_CT_(*BA*) = 0.426*e*). The electron-attraction character of fragment *C* in the NMU-DCP becomes so prominent that it draws electron density not only from fragment *A* but also significantly from fragment *B*. Consequently, *q*_CT_(*CB*) (0.174*e*) emerges as the second most significant CT pathway during the emission. Similarly, the enhanced electron-attraction character of fragment *C* produces a slight LE character on it (*q*_LE_(*C*) = 0.058*e*), in constrast with NMU-1, which was on fragment *A*. Regarding the DCP simulant (fragment *D*), its electron-attracting nature results in CT toward the fragment *A* electron-donor during fluorescence, but it is in a very small proportion (*q*_CT_(*DA*) = 0.031*e*).

Upon hydration of NMU-DCP, the DCP group is eliminated, yielding a protonated pyridine moiety (fragment *C*)—see the NMU+H molecule in Table 2c. Similarly to the NMU-DCP molecule, the positively charged nitrogen in fragment *C* of NMU+H induces a greater CT from both fragments *A* and *B* toward fragment *C* in emission (*q*_CT_(*CA*) = 0.540*e* and *q*_CT_(*CB*) = 0.119*e*). Therefore, the emission of NMU+H exhibits a CT pattern similar to that of the NMU-DCP. However, substituting the strongly electronegative DCP group with a proton attenuates the electron-withdrawing capacity of fragment *C*, thereby reducing the CT intensity. In both NMU+H and NMU-DCP cases, the predominant emission pathways involve CT from fragment *C*to fragments *A* and *B*. Nonetheless, as previously discussed, these contributions are diminished in NMU+H (0.540*e* and 0.119*e*, respectively) compared to NMU-DCP (0.666*e* and 0.174*e*).

In the absence of the electronegative DCP molecule in the NMU+H system, alternative electronic effects become predominant. For example, the local excitation within fragment A (*q*_LE_(*A*)) is now equally significant as *q*_CT_(*CB*), both with values of approximately 0.12*e*. Additionally, as fragment *C* (the positive pyridine group) in NMU+H becomes less electronegative than in NMU-DCP, the effects of the ester group (fragment B) become more pronounced. This is clear for the CT from fragment *B* to fragment *A* during fluorescence, with *q*_CT_(*CB*) values of 0.023*e* for NMU-DCP and 0.113*e* for NMU+H. Therefore, fragment *B* recovers its importance with fragment *C* in terms of electron-accepting character. Consequently, due to the emission, the electron density moves from the electron-acceptor fragment *B* toward the electron-donor fragment *A*.

For NMU-1, no vibrational modes display significant HR factors (Figure 8a), resulting in strong fluorescence with a computed intensity more than twice that of HBQ-AE. This reflects a regime of weak vibronic coupling. When the target is present, the HBQ-DCP system shows 13 vibrational modes with HR > 0.3, one with HR > 1.0 at 1665 cm^–1^—see Figure 8b. This marked increase in vibrational modes with significant HR factors, especially at low-to-medium frequency, results in a broadened spectrum of negligible intensity. After hydrolysis, the resulting NMU+H molecule (Figure 8c) exhibits a single vibrational mode with HR > 0.3 (1677 cm^–1^, with the scissoring of the pyridine ring members (fragment C)). While less pronounced than in NMU-DCP, this mode is sufficient to dramatically decrease fluorescence intensity.

**Figure 8:**
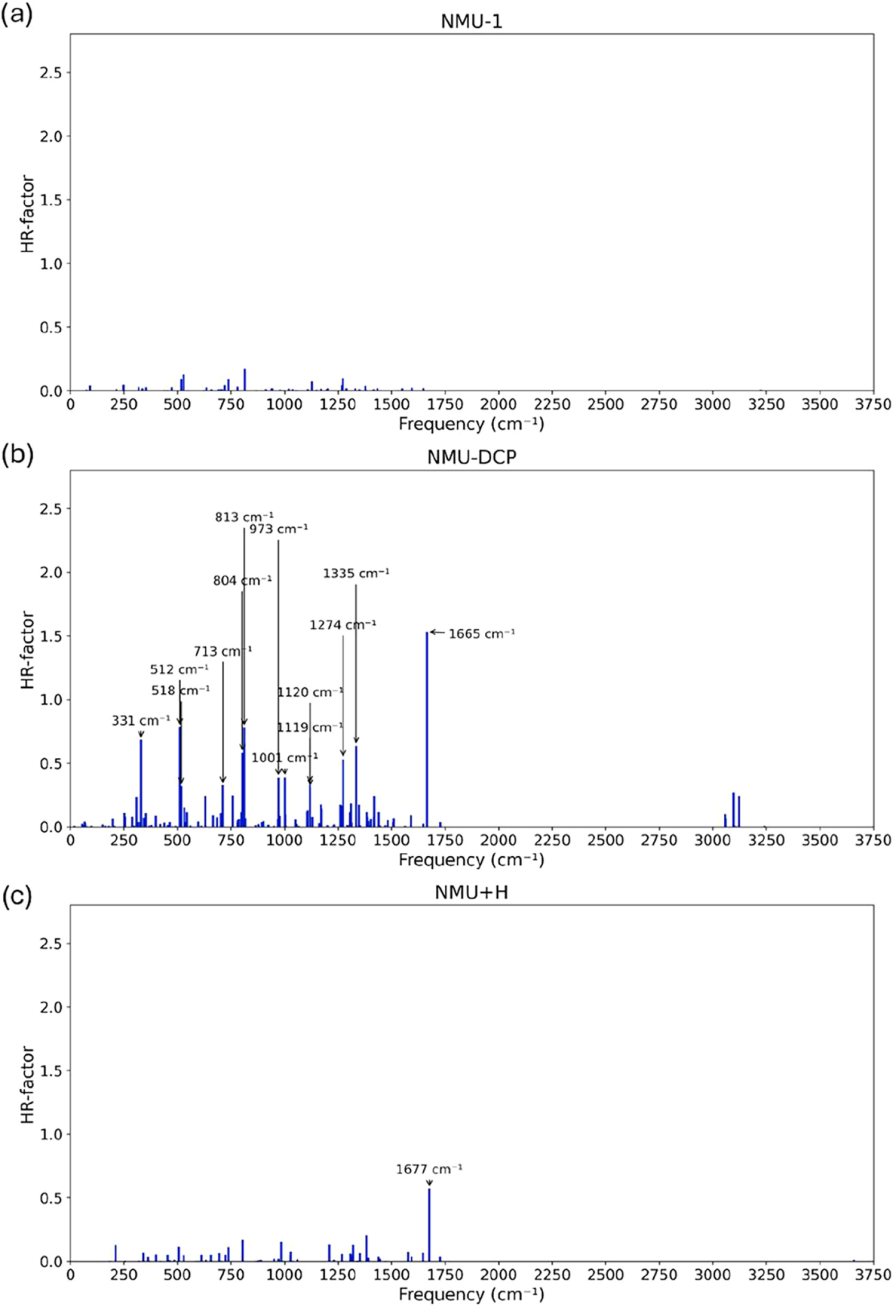
Calculated *S*_1_-*S*_0_ Huang–Rhys factors from of the NMU system, namely: (a) NMU-1, (b) NMU-DCP, and (c) NMU+H. The vibrational modes with large contributions to the Huang–Rhys factor are shown.

## Conclusions

We used DFT and a path integral method to compute the fluorescence spectra with vibronic effects of the main compounds involved in the fluorescent mechanism of the HBQ-AE and NMU-1 probes, which were synthesized for detecting nerve agents (NAs). The charge transfer (CT) effects, which play an important role in the fluorescence mechanism, were comprehensively characterized using the natural transition orbitals (NTOs). The DCP NA simulant was used for the investigations.

For the HBQ systems (HBQ-AE, HBQ-DCP, HBQ+H, and HBQ-Keto), the experimental group indicated an increase in the fluorescence intensity upon binding to the DCP target, attributing this enhancement to the formation of the HBQ-DCP product.^36^ However, our theoretical calculations revealed that HBQ-DCP exhibits almost negligible fluorescence compared to the other HBQ-based molecules, reducing by approximately 99.65% the HBQ-AE fluorescence intensity. This suggests that the DCP target likely acts as a fluorescence quencher of the HBQ-AE probe. Given that the experimental probe solution was prepared in water, hydrolysis of the HBQ-DCP product becomes plausible. Considering that the fluorescence peak of the hydrolysis product HBQ+H differs from the experimental one by only 0.23 eV, the true detection mechanism of the DCP target involves the hydrolysis of the HBQ-DCP product, rather than emission from the HBQ-DCP itself as previously assumed.^36^ In the case of acetylcholinesterase (AChE) detection, our theoretical results agree with the experimental observations, which attribute the fluorescence generation to the formation of a carbonyl-containing product (HBQ-Keto).

For the NMU systems (NMU-1, NMU-DCP, and NMU+H), we confirmed the experimental result that the mechanism of the fluorescent probe NMU-1 for detecting the DCP simulant works through fluorescence quenching. In particular, we quantified a fluorescence intensity reduction of approximately 99.95% in the fluorescence intensity of NMU-1 bonded to DCP (the NMU-DCP molecule). The hydrolysis product NMU+H also displayed a very weak, nearly negligible fluorescence signal, which explains the near-complete fluorescence suppression observed by the experimental group.

The hydrolysis product HBQ+H retains significant fluorescence (only a 49.93% reduction relative to HBQ-AE), whereas NMU+H shows markedly lower emission (94.81% decrease). Consequently, HBQ-AE acts as a fluorescence turn-on sensor via its emissive hydrolysis product, HBQ+H, rather than the emission quenching by the HBQ-DCP product as previously thought. In contrast, NMU-1 is a turn-off sensor, as both NMU-DCP and NMU+H are essentially nonemissive.

The theoretical approach combining DFT and a path integral method to compute the fluorescence spectra showed excellent agreement with the experimental data, with blue shift deviations ranging from 0.12 to 0.24 eV. These small discrepancies highlight the balance between computational cost and accuracy offered by the theoretical approach, which has proven to be sufficiently accurate for reproducing and interpreting the fluorescence spectra of the investigated systems.

Our comprehensive investigation of CT showed its effect in modulating the emission intensities in the fluorescence spectra of both probes. When the fluorescent probe (HBQ-AE or NMU-1) interacts with the target molecule, the DCP simulant, the fluorescence is quenched in both formed products (HBQ-DCP and NMU-DCP) due to an enhanced CT within the probes resulting from the electron-accepting nature of DCP and the formation of a positively charged nitrogen atom in the probe. Hydration should proceed as the experimental probe-target system is formed in a water solution. Following hydration and the elimination of the DCP simulant, a hydrated compound is formed in both cases, exhibiting intermediate properties between the isolated probe and the probe bonded to DCP. This suggests that the hydrated probe (probe+H) induces a greater CT than the free probe, thereby reducing the fluorescence spectrum’s intensity. The DCP simulant further amplifies CT effects, resulting in almost complete fluorescence quenching in both cases.

For the HBQ group members, fluorescence is governed by the transfer of electron density from the pyridine-based unit to the naphthalene-based unit. In the NMU-1 molecule, fluorescence is mainly driven by the return of electron density from the ester group to the julolidine-based unit. Upon target detection and formation of the NMU-DCP molecule, a strong CT from the pyrene-based unit to the julolidine-based unit occurs during the emission process. This CT is slightly attenuated after hydrolysis and formation of NMU+H. These results indicate that the presence of the DCP target significantly enhances the CT character in the probe-target product compared to the free probe, while the hydrolyzed probe (NMU+H) and HBQ-Keto species exhibit intermediate CT behavior.

The analysis of the Huang–Rhys factors showed that in systems where charge is transfer is enhanced, strong vibronic coupling, particularly involving low-frequency modes, provides efficient nonradiative decay channels, thereby suppressing emission. Conversely, when HR factors are small, an intense fluorescence is preserved.

## Supplementary Material



## Data Availability

The data that support the findings of this study, including Orca input and outputs, are available in the Supporting Information of this article. Additional data are available in GitHub and Zenodo and can be accessed via 10.5281/zenodo.17038816.

## Notes

Section 1 presents the coordinates and CT decomposition for the investigated molecules. Table S1 lists the B3LYP/def2-TZVP­(-f)/CPCM­(water) Cartesian coordinates of the optimized *S*_0_ and *S*_1_ strucutures; The *S*_0_ optimized structures are shown in Figure S1 to Figure S7; Table S2 presents the energy, oscillator strength, and CT decomposition pathways for HBQ-AE and HBQ-DCP; Section 2 presents the test calculations to validate the chosen theoretical level for geometry optimizations and CT decomposition (PDF)
