# Supplementary material for: Theoretical Investigation of Charge Modulation Effects in Two Pyridine-Based Fluorescence Probes for Nerve Agent and Acetylcholinesterase (AChE) Detection
Source: ACS Omega. 2025 Oct 3;10(40):47266–80. doi: 10.1021/acsomega.5c06101 (PMC12529199; doi:10.1021/acsomega.5c06101)
Supplement: Supplementary file 1 [file ao5c06101_si_001.pdf]

Supporting Information

Theoretical investigation of charge modulation effects in two pyridine-based fluorescence probes for nerve agent and acetylcholinesterase (AChE) detection

Matheus Máximo-Canadas[a], Bruno Gabriel Motta Rodrigues[a], Itamar Borges Jr[a,*]

[a]*Departamento de Química, Instituto Militar de Engenharia (IME), Praça General Tibúrcio, 80, Rio de Janeiro, RJ 22290-270, Brasil*

[*]Email: itamar@ime.eb.br

1. Coordinates and CT decomposition

Table S1: B3LYP/def2-TZVP(-f)/CPCM(water) Cartesian coordinates of the optimized molecules.

HBQ-AE (S_0)				***HBQ-AE*** (S_1)			
C	-3.346738	-0.786771	-0.324041	C	-3.302758	-1.102259	-0.236082
C	-2.896629	-1.711282	-1.271432	C	-2.756979	-2.043749	-1.107194
C	-1.563461	-1.744452	-1.601973	C	-1.425083	-1.919625	-1.474444
C	-2.470828	0.091697	0.274571	C	-2.521505	-0.059644	0.281089
C	-1.086073	0.096220	-0.032327	C	-1.121232	0.041711	0.026868
O	-3.017534	0.897191	1.268766	O	-3.222768	0.811078	1.061510
C	-0.647893	-0.860761	-0.997385	C	-0.597939	-0.898446	-0.941169
C	0.736050	-0.939874	-1.357364	C	0.744081	-0.816714	-1.325267
C	1.659167	-0.125879	-0.797207	C	1.585698	0.143500	-0.769293
C	-0.090921	0.980199	0.560046	C	-0.226144	0.940764	0.694139
C	1.271067	0.849173	0.171121	C	1.145358	1.012393	0.245593
N	-0.467192	1.912445	1.464427	N	-0.668883	1.698360	1.732491
C	0.448699	2.701669	1.997645	C	0.183292	2.595572	2.272234
C	1.812355	2.639293	1.682801	C	1.494610	2.783982	1.856078
C	2.218072	1.705904	0.760691	C	2.003073	1.967302	0.843375
C	-3.332690	2.191008	0.942432	C	-3.216723	2.172320	0.770046
O	-3.243087	2.617718	-0.199030	O	-2.851830	2.590191	-0.312156
N	-3.763759	2.885857	2.012337	N	-3.768101	2.885479	1.764910
C	-3.903233	2.322868	3.350670	C	-4.053289	2.357931	3.094761
C	-4.283135	4.234588	1.830130	C	-3.876279	4.331844	1.615013
H	-5.360090	4.257168	2.018335	H	-3.099638	4.838124	2.194632
H	-4.090392	4.565537	0.815491	H	-4.851168	4.655526	1.980698
H	-3.794558	4.912943	2.531879	H	-3.774552	4.600670	0.569016
H	-3.278175	1.444919	3.463653	H	-3.260306	2.634862	3.794791
H	-4.943613	2.053089	3.554049	H	-4.143435	1.278389	3.069969
H	-3.593289	3.073727	4.078484	H	-4.992621	2.786080	3.445302
H	-1.193450	-2.455868	-2.329509	H	-0.985678	-2.619395	-2.174247
H	-4.390153	-0.746498	-0.040597	H	-4.352408	-1.121360	0.024118
H	-3.598185	-2.393217	-1.733972	H	-3.372345	-2.833085	-1.515201
H	1.026423	-1.679204	-2.093548	H	1.125663	-1.512225	-2.061632
H	2.704721	-0.195576	-1.071809	H	2.612320	0.216485	-1.107706
H	0.093625	3.433395	2.716697	H	-0.209137	3.176841	3.100405
H	2.513591	3.314029	2.155117	H	2.112806	3.525709	2.344657
H	3.259461	1.616582	0.475232	H	3.032062	2.039220	0.517078
HBQ-DCP (S_0)				***HBQ-DCP*** (S_1)			
C	-3.393728	-0.957296	1.568122				
C	-3.200013	-1.868576	2.611714	C	-4.794164	0.324572	0.452536
C	-2.005184	-1.910819	3.290914	C	-5.400318	-0.529476	1.368699
C	-2.421484	-0.025928	1.271977	C	-4.615765	-1.113941	2.336022
C	-1.215169	0.038378	2.024222	C	-3.424868	0.633753	0.540116
O	-2.469362	0.766933	0.152646	C	-2.631205	0.201248	1.625080
C	-0.980133	-1.004475	2.973207	O	-2.948858	1.345147	-0.512607
C	0.327343	-1.200693	3.518154	C	-3.224669	-0.789144	2.460916
C	1.380003	-0.480802	3.071251	C	-2.435357	-1.476764	3.396947
C	-0.147492	0.976911	1.803961	C	-1.083208	-1.223516	3.477154

C	1.172119	0.622744	2.188510	C	-1.290752	0.611624	1.906349
N	-0.314723	2.231531	1.229264	C	-0.480113	-0.174939	2.730327
C	0.748099	2.912045	0.738798	N	-0.733872	1.799471	1.351138
C	2.041113	2.464160	0.889902	C	0.662851	1.837519	1.219585
C	2.253509	1.366527	1.699114	C	1.474959	0.983843	1.916061
C	-3.664030	1.047367	-0.508139	C	0.925649	0.038184	2.777977
O	-4.718293	1.181014	0.084776	C	-1.893590	0.842277	-1.292181
N	-3.466123	1.188424	-1.826439	O	-1.338440	-0.199909	-1.021851
C	-2.199252	0.963637	-2.517430	N	-1.641921	1.653641	-2.328923
C	-4.572337	1.628261	-2.670205	C	-2.451899	2.807870	-2.710798
H	-4.829061	0.843177	-3.384337	C	-0.624415	1.246436	-3.293769
H	-4.275172	2.520713	-3.223934	H	-1.094389	0.817449	-4.182398
H	-5.436024	1.854361	-2.054828	H	0.035723	0.512035	-2.845082
H	-1.784074	1.913602	-2.861297	H	-0.051964	2.125249	-3.588598
H	-2.382460	0.329730	-3.386358	H	-1.788404	3.647947	-2.915250
H	-1.485909	0.474353	-1.865864	H	-3.137177	3.081544	-1.919469
H	-1.818868	-2.669278	4.039749	H	-3.012859	2.576323	-3.619282
H	-4.295738	-0.999545	0.979100	H	-5.033147	-1.829892	3.031585
H	-3.982970	-2.579936	2.840060	H	-5.346937	0.735282	-0.381481
H	0.466714	-2.001028	4.233280	H	-6.452791	-0.759797	1.288075
H	2.391649	-0.697441	3.387784	H	-2.888432	-2.238374	4.017294
H	0.533193	3.861066	0.271969	H	-0.458729	-1.811598	4.137501
H	2.854557	3.028777	0.459756	H	1.048167	2.624762	0.590320
H	3.258943	1.062396	1.961709	H	2.545657	1.080474	1.799914
P	-1.724483	3.288371	1.598286	H	1.540475	-0.573071	3.421509
O	-2.697530	2.610294	2.453380	P	-1.499292	3.332017	1.597763
O	-2.070836	3.723700	0.139616	O	-2.933156	3.197880	1.893501
O	-1.039156	4.558986	2.217278	O	-1.047642	4.042072	0.269185
C	-2.965152	4.867654	-0.149961	O	-0.652454	4.089697	2.700071
C	-0.990933	4.839831	3.665079	C	-1.222254	5.487960	0.068172
C	-2.692659	5.322268	-1.560618	C	-1.066265	4.194574	4.103195
H	-2.758297	5.652192	0.575664	C	-0.189863	5.956727	-0.927160
H	-3.984328	4.508496	-0.021857	H	-1.107057	5.990241	1.027567
H	-1.665354	5.671393	-1.667845	H	-2.237353	5.636916	-0.296874
H	-3.365030	6.149454	-1.795922	H	0.819749	5.799169	-0.546198
H	-2.874234	4.517938	-2.273653	H	-0.329339	7.024911	-1.103726
C	0.407406	4.635525	4.196005	H	-0.296483	5.434839	-1.878522
H	-1.719234	4.202025	4.160743	C	-0.144121	3.387218	4.986171
H	-1.308707	5.876891	3.745674	H	-2.101370	3.869146	4.186890
H	0.422887	4.914556	5.251575	H	-1.011184	5.257438	4.331946
H	1.124026	5.262671	3.664805	H	0.890875	3.711181	4.870472
H	0.715662	3.592529	4.113492	H	-0.209356	2.322768	4.758483
				H	-0.434989	3.534080	6.028498
HBQ-H (S_0)				***HBQ-H (S_1)***			
C	-3.01703	-0.84514	0.12957	C	-3.07574	-0.63751	-0.03928
C	-2.56834	-1.87168	-0.70651	C	-2.66486	-1.72392	-0.80071
C	-1.27466	-1.86755	-1.17360	C	-1.34031	-1.81027	-1.16923
C	-2.17077	0.17992	0.48391	C	-2.16890	0.37170	0.34590
C	-0.82605	0.21740	0.03943	C	-0.79014	0.31906	0.00090
O	-2.61701	1.16244	1.35850	O	-2.60259	1.38036	1.13824
C	-0.38625	-0.84007	-0.81255	C	-0.39127	-0.81095	-0.78018

C	0.95633	-0.85677	-1.30841	C	0.94125	-0.95247	-1.18588
C	1.84362	0.11408	-0.99362	C	1.88338	-0.00890	-0.82594
C	0.13998	1.23032	0.37663	C	0.20437	1.28547	0.35651
C	1.45820	1.18821	-0.13667	C	1.54148	1.12816	-0.05207
N	-0.16520	2.26560	1.20771	N	-0.09503	2.41470	1.10797
C	0.68196	3.23670	1.55150	C	0.85317	3.34874	1.45326
C	1.97461	3.23550	1.05800	C	2.15507	3.20148	1.06384
C	2.35593	2.20822	0.21948	C	2.52980	2.08820	0.30041
C	-3.76259	1.89746	1.00842	C	-3.89495	1.94443	0.94342
O	-4.17709	1.91832	-0.13305	O	-4.40722	1.93343	-0.15483
N	-4.25744	2.55607	2.06303	N	-4.35567	2.48046	2.07218
C	-5.36110	3.49085	1.86226	C	-5.59899	3.24891	2.03311
C	-3.77426	2.43270	3.43703	C	-3.69024	2.42028	3.37375
H	-3.11276	1.58074	3.53888	H	-2.88911	1.69106	3.36713
H	-4.63270	2.29543	4.09583	H	-4.42645	2.13167	4.12432
H	-3.24717	3.34145	3.73593	H	-3.29005	3.40227	3.63331
H	-5.57136	3.58850	0.80286	H	-5.99407	3.25771	1.02337
H	-5.08752	4.46518	2.27082	H	-5.40327	4.27165	2.35981
H	-6.25322	3.13117	2.37883	H	-6.32649	2.79561	2.70785
H	-0.91968	-2.65760	-1.82220	H	-0.98232	-2.64524	-1.75674
H	-4.03119	-0.84753	0.50372	H	-4.10051	-0.56404	0.29250
H	-3.24697	-2.66907	-0.97896	H	-3.37348	-2.48917	-1.08274
H	1.24549	-1.67624	-1.95394	H	1.22672	-1.80844	-1.78226
H	2.85595	0.09800	-1.37511	H	2.91329	-0.12980	-1.13702
H	0.29621	3.99102	2.22108	H	0.49182	4.18157	2.03618
H	2.65609	4.02473	1.33782	H	2.87670	3.95328	1.34964
H	3.36268	2.17396	-0.17767	H	3.54771	1.94618	-0.03055
H	-1.10563	2.27848	1.59286	H	-1.04206	2.55510	1.41565
HBQ-Keto (S_0)				***HBQ-Keto (S_1)***			
C	-3.63957	-0.54618	0.13739	C	-3.69175	-0.58909	0.13541
C	-3.64926	-1.93306	0.09291	C	-3.68760	-1.97875	0.08937
C	-2.47298	-2.67520	0.04405	C	-2.49861	-2.67546	0.04403
C	-1.24213	-2.01156	0.03833	C	-1.23706	-1.99145	0.04013
C	-1.20895	-0.58712	0.08288	C	-1.20940	-0.58072	0.08532
C	-2.43053	0.19075	0.13464	C	-2.48019	0.16557	0.13771
O	-2.40814	1.48210	0.17623	O	-2.51459	1.43439	0.18479
C	0.06130	0.04680	0.07476	C	0.07037	0.06582	0.07703
C	1.27767	-0.68254	0.02508	C	1.26442	-0.67274	0.02667
C	1.20240	-2.11101	-0.01879	C	1.18593	-2.08249	-0.01585
C	-0.00147	-2.73536	-0.01199	C	-0.02365	-2.72931	-0.00970
N	0.12095	1.40523	0.11653	N	0.14517	1.43494	0.11737
C	1.26569	2.09171	0.11370	C	1.32348	2.11828	0.11238
C	2.47614	1.42419	0.06658	C	2.52099	1.41999	0.06379
C	2.47676	0.03578	0.02235	C	2.52008	0.03963	0.02042
H	-0.85543	1.82300	0.15177	H	-0.75781	1.91238	0.15452
H	-4.56900	0.00930	0.17575	H	-4.61837	-0.03111	0.17291
H	-4.60167	-2.45118	0.09691	H	-4.62628	-2.51898	0.08986
H	-2.50006	-3.75668	0.01021	H	-2.49570	-3.75726	0.00948
H	-0.04984	-3.81739	-0.04546	H	-0.07143	-3.80986	-0.04299
H	2.12481	-2.67662	-0.05700	H	2.10791	-2.65008	-0.05399

H	3.41469	-0.50479	-0.01454	H	3.44442	-0.51983	-0.01853
H	1.18309	3.16850	0.15001	H	1.25436	3.19420	0.14689
H	3.39818	1.98666	0.06500	H	3.44796	1.97726	0.06027
NMU-1 (S_0)				***NMU-1 (S_1)***			
C	-6.55690	-1.50175	-0.08448	C	-6.56158	-1.48120	-0.14331
C	-6.55861	-2.65952	-1.06738	C	-6.53799	-2.59280	-1.17814
C	-5.55820	-3.71340	-0.60891	C	-5.56489	-3.67781	-0.73465
H	-6.29409	-2.29283	-2.06285	H	-6.23591	-2.18303	-2.14551
H	-7.56387	-3.07926	-1.12680	H	-7.54591	-2.99426	-1.28865
C	-4.20401	-3.10116	-0.33703	C	-4.21951	-3.09144	-0.39294
H	-5.45850	-4.50935	-1.34884	H	-5.44609	-4.43922	-1.50718
H	-5.93417	-4.18337	0.30664	H	-5.97276	-4.18717	0.14627
C	-4.09927	-1.71974	0.03026	C	-4.11421	-1.72973	0.03305
N	-5.21889	-0.93347	0.07389	N	-5.22927	-0.93876	0.10323
H	-6.94380	-1.83408	0.88844	H	-6.97583	-1.85910	0.80373
H	-7.20948	-0.70106	-0.43457	H	-7.19497	-0.65460	-0.46473
C	-5.16265	0.41102	0.64357	C	-5.17302	0.39683	0.68641
C	-3.86751	1.11077	0.27098	C	-3.86424	1.09157	0.35623
H	-5.26713	0.36322	1.73606	H	-5.29477	0.31999	1.77761
H	-6.02028	0.96388	0.25972	H	-6.02522	0.95714	0.30380
C	-2.67301	0.28232	0.72989	C	-2.69119	0.24120	0.82674
H	-3.84488	2.10038	0.72947	H	-3.84708	2.06889	0.83952
H	-3.83401	1.24936	-0.81279	H	-3.80484	1.25444	-0.72279
C	-2.82704	-1.16589	0.33052	C	-2.83671	-1.18890	0.37899
H	-2.57228	0.35390	1.81844	H	-2.63199	0.26947	1.92121
H	-1.74936	0.68428	0.31251	H	-1.74856	0.64710	0.46079
C	-3.06636	-3.86393	-0.42406	C	-3.07793	-3.85567	-0.47759
C	-1.72107	-1.99366	0.24209	C	-1.73244	-2.02094	0.29773
C	-1.79139	-3.34138	-0.13890	C	-1.79918	-3.35722	-0.13537
O	-0.50125	-1.44611	0.55060	O	-0.52502	-1.47721	0.66469
C	0.69074	-2.14368	0.49735	C	0.67957	-2.18008	0.59720
C	-0.59034	-4.08268	-0.19987	C	-0.60339	-4.12013	-0.20256
C	0.62959	-3.53709	0.10105	C	0.64186	-3.55646	0.14950
O	1.68998	-1.50683	0.78187	O	1.65787	-1.51755	0.91737
C	1.87953	-4.31758	0.00508	C	1.87119	-4.31566	0.04646
C	2.90133	-4.24011	0.95637	C	3.07752	-3.99764	0.71543
C	2.08038	-5.19847	-1.06510	C	1.91452	-5.49237	-0.75338
N	3.16100	-5.96592	-1.21577	N	2.98126	-6.26913	-0.90339
C	4.11843	-5.88021	-0.28696	C	4.10337	-5.93869	-0.24908
C	4.03043	-5.03090	0.80872	C	4.18373	-4.80980	0.56606
H	1.32979	-5.27330	-1.84514	H	1.04217	-5.78782	-1.32288
H	4.83116	-4.99563	1.53573	H	5.10627	-4.58071	1.08425
H	4.98883	-6.51199	-0.42716	H	4.95672	-6.59461	-0.38023
H	2.80646	-3.57683	1.80411	H	3.12477	-3.12976	1.35245
H	-3.14600	-4.90579	-0.71375	H	-3.15601	-4.88322	-0.81353
H	-0.65196	-5.12748	-0.48256	H	-0.67897	-5.15930	-0.48233
NMU-DCP (S_0)				***NMU-DCP (S_1)***			
C	-6.46986	-1.53447	-0.09936	C	-6.38001	-1.42640	0.18965

C	-6.42172	-2.80153	-0.93443	C	-6.48404	-2.92851	0.02419
C	-5.39199	-3.75738	-0.34573	C	-5.41736	-3.60448	0.87244
H	-6.15922	-2.54950	-1.96535	H	-6.35806	-3.19244	-1.02833
H	-7.41211	-3.25849	-0.94847	H	-7.48168	-3.24336	0.32888
C	-4.06037	-3.07146	-0.15437	C	-4.05964	-3.04860	0.55643
H	-5.26234	-4.63545	-0.98026	H	-5.41173	-4.68449	0.72709
H	-5.75535	-4.12043	0.62198	H	-5.63087	-3.42654	1.93268
C	-4.00568	-1.64926	0.04144	C	-3.92486	-1.68165	0.09486
N	-5.14993	-0.91080	0.01380	N	-5.01974	-0.92590	-0.07251
H	-6.86336	-1.75659	0.90092	H	-6.64874	-1.12397	1.20728
H	-7.13610	-0.80064	-0.55388	H	-7.04378	-0.90399	-0.49608
C	-5.14149	0.49899	0.40496	C	-4.93050	0.50306	-0.41451
C	-3.88186	1.19525	-0.07843	C	-3.74051	0.79049	-1.30707
H	-5.22814	0.58312	1.49596	H	-4.86221	1.06251	0.52402
H	-6.02678	0.96345	-0.02849	H	-5.86540	0.77469	-0.89744
C	-2.64901	0.47896	0.45980	C	-2.46503	0.29010	-0.64514
H	-3.89344	2.23511	0.25075	H	-3.68521	1.86422	-1.48313
H	-3.86925	1.19597	-1.17136	H	-3.88410	0.30216	-2.27342
C	-2.75089	-1.01315	0.25280	C	-2.62705	-1.12495	-0.16853
H	-2.53541	0.69346	1.52790	H	-2.21854	0.92400	0.21414
H	-1.74833	0.85729	-0.02444	H	-1.61988	0.35234	-1.32881
C	-2.89873	-3.79660	-0.14849	C	-2.93174	-3.79791	0.72023
C	-1.61907	-1.80421	0.26494	C	-1.53666	-1.93687	0.03548
C	-1.64170	-3.19538	0.06429	C	-1.64751	-3.27338	0.46944
O	-0.41910	-1.17891	0.49216	O	-0.30390	-1.40011	-0.20708
C	0.79381	-1.83451	0.53105	C	0.86684	-2.11480	-0.07871
C	-0.42434	-3.89608	0.10208	C	-0.46055	-4.03714	0.62471
C	0.77773	-3.26875	0.32893	C	0.76064	-3.50882	0.35864
O	1.77383	-1.13659	0.72196	O	1.89446	-1.53577	-0.33941
C	2.05020	-4.00465	0.35730	C	2.01213	-4.30199	0.49043
C	3.11131	-3.66977	1.21581	C	2.90710	-4.05643	1.55457
C	2.24381	-5.09325	-0.48157	C	2.25017	-5.28886	-0.41737
N	3.38770	-5.81449	-0.45818	N	3.40097	-6.09765	-0.32320
C	4.40467	-5.49374	0.37064	C	4.29431	-5.86371	0.74056
C	4.28021	-4.41278	1.21563	C	4.05327	-4.88153	1.64274
H	1.49994	-5.40413	-1.19955	H	1.58890	-5.49627	-1.24208
H	5.09706	-4.16557	1.87799	H	4.76664	-4.74090	2.44298
H	5.28148	-6.11941	0.32056	H	5.15371	-6.51044	0.77141
H	3.01005	-2.83117	1.88718	H	2.71700	-3.27905	2.27602
H	-2.93910	-4.86877	-0.30412	H	-3.01330	-4.82334	1.05734
H	-0.45632	-4.97137	-0.03281	H	-0.54503	-5.06493	0.95522
P	3.55664	-7.24175	-1.54195	P	3.67416	-7.32658	-1.45949
O	4.92068	-7.74774	-1.37798	O	4.93016	-8.03001	-1.15106
O	3.07311	-6.69786	-2.91938	O	3.53961	-6.64865	-2.88073
O	2.33641	-8.12843	-1.14364	O	2.34740	-8.18334	-1.51440
C	2.35622	-8.98729	0.06200	C	1.99980	-9.06799	-0.39904
C	3.96653	-5.95810	-3.84306	C	4.58174	-5.73463	-3.35663
C	1.02607	-9.69084	0.14043	C	0.71149	-9.77249	-0.74787
H	2.52856	-8.34552	0.92557	H	1.89199	-8.45910	0.49944
H	3.18501	-9.68359	-0.04840	H	2.81781	-9.77362	-0.25711
H	3.35090	-5.13790	-4.20291	H	4.67729	-4.91923	-2.63853
C	4.41849	-6.86757	-4.95768	C	4.17023	-5.22612	-4.71678
H	4.80298	-5.55336	-3.27508	H	5.52117	-6.28489	-3.39980

H	0.20897	-8.97502	0.23317	H	-0.09772	-9.05647	-0.89600
H	1.02330	-10.33411	1.02226	H	0.43682	-10.43840	0.07210
H	0.86042	-10.31200	-0.74020	H	0.82712	-10.36991	-1.65300
H	3.56361	-7.27498	-5.49819	H	3.22566	-4.68372	-4.66172
H	5.02515	-7.68899	-4.57590	H	4.06683	-6.04842	-5.42578
H	5.02390	-6.28752	-5.65716	H	4.93774	-4.54499	-5.08875
NMU-H (S_0)				***NMU-H (S_1)***			
C	-6.62302	-1.49584	-0.05520	C	-6.61769	-1.50488	0.05146
C	-6.60645	-2.60519	-1.09171	C	-6.64066	-2.71894	-0.85442
C	-5.59574	-3.66718	-0.67812	C	-5.61979	-3.73435	-0.36345
H	-6.34214	-2.18991	-2.06786	H	-6.41555	-2.41771	-1.88011
H	-7.60680	-3.03222	-1.17460	H	-7.64535	-3.14057	-0.84485
C	-4.24750	-3.05030	-0.39355	C	-4.26611	-3.10066	-0.22848
H	-5.49251	-4.43459	-1.44682	H	-5.55481	-4.59476	-1.02891
H	-5.96126	-4.17219	0.22282	H	-5.92887	-4.11252	0.61784
C	-4.16066	-1.68552	0.04702	C	-4.15417	-1.68849	0.07637
N	-5.28872	-0.93026	0.15552	N	-5.26371	-0.94745	0.20966
H	-7.02075	-1.87325	0.89562	H	-6.98701	-1.75468	1.05174
H	-7.27189	-0.68073	-0.37673	H	-7.24183	-0.70450	-0.34046
C	-5.25114	0.39791	0.76887	C	-5.20854	0.45726	0.64677
C	-3.97000	1.13225	0.41692	C	-3.96438	1.15200	0.13183
H	-5.35166	0.30715	1.85807	H	-5.23774	0.46365	1.74115
H	-6.11980	0.94756	0.40696	H	-6.11280	0.93978	0.28535
C	-2.76067	0.30373	0.83379	C	-2.72565	0.38233	0.56617
H	-3.96033	2.10145	0.91720	H	-3.94159	2.16811	0.52422
H	-3.94566	1.31694	-0.66008	H	-4.00924	1.21710	-0.95750
C	-2.89118	-1.12313	0.35855	C	-2.86527	-1.08050	0.25511
H	-2.66255	0.31916	1.92475	H	-2.57328	0.49966	1.64493
H	-1.84405	0.74225	0.43895	H	-1.83252	0.77585	0.08313
C	-3.10115	-3.78509	-0.53606	C	-3.12380	-3.83444	-0.35758
C	-1.77506	-1.92236	0.20765	C	-1.76104	-1.89039	0.13501
C	-1.82910	-3.25283	-0.24257	C	-1.84743	-3.26207	-0.17893
O	-0.55877	-1.37071	0.52206	O	-0.53925	-1.31151	0.32760
C	0.64264	-2.04081	0.41216	C	0.64783	-2.00496	0.22028
C	-0.62582	-3.96741	-0.35994	C	-0.64499	-4.00638	-0.30282
C	0.59427	-3.41293	-0.04991	C	0.56994	-3.42780	-0.12194
O	1.63772	-1.40218	0.70507	O	1.66371	-1.37967	0.41201
C	1.84849	-4.16527	-0.19530	C	1.83773	-4.19171	-0.24402
C	2.95494	-3.99836	0.65488	C	2.48613	-4.68247	0.92119
C	1.98025	-5.10747	-1.20979	C	2.35720	-4.42624	-1.48153
N	3.10703	-5.82219	-1.33690	N	3.53961	-5.13835	-1.61552
C	4.17110	-5.68064	-0.53067	C	4.20045	-5.63297	-0.49631
C	4.10736	-4.75217	0.48564	C	3.68981	-5.41364	0.74269
H	1.21494	-5.30504	-1.94483	H	1.90326	-4.07603	-2.39459
H	4.95302	-4.62791	1.14609	H	4.22052	-5.80773	1.59834
H	5.02338	-6.31080	-0.73306	H	5.10989	-6.17938	-0.68624
H	2.90333	-3.27895	1.45717	H	2.07490	-4.50628	1.90145
H	-3.16511	-4.81192	-0.87795	H	-3.18752	-4.88917	-0.59293
H	-0.68211	-4.99897	-0.68838	H	-0.71043	-5.05924	-0.54789
H	3.15929	-6.49849	-2.09285	H	3.90621	-5.31966	-2.53292

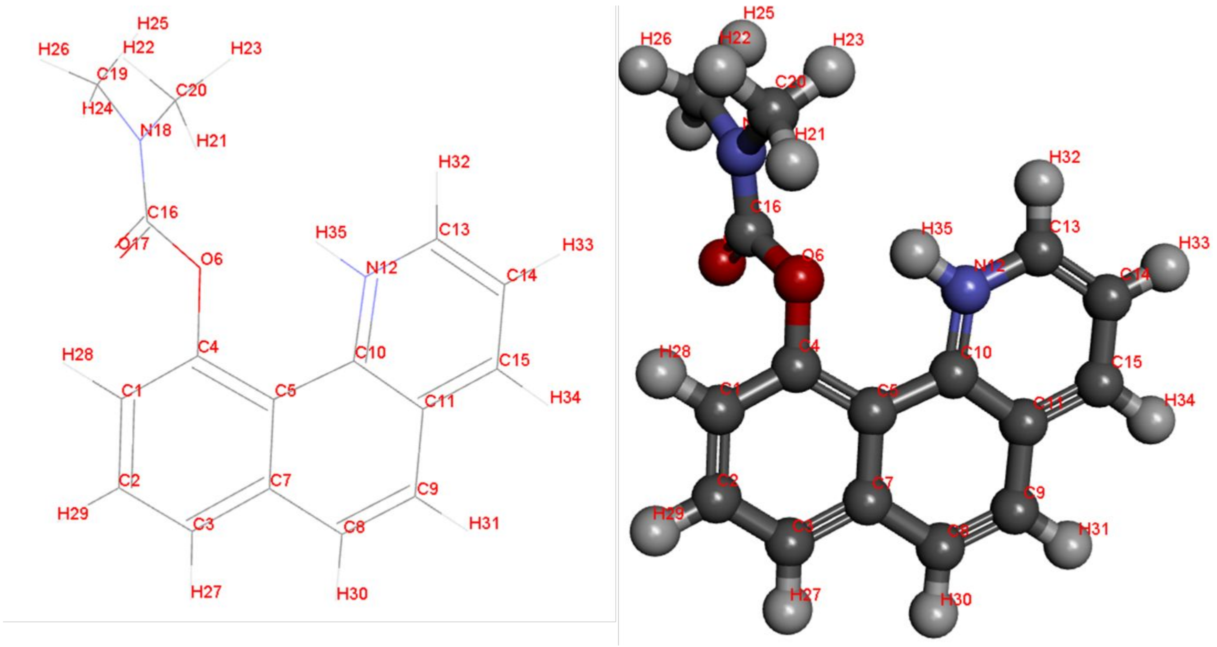

Figure S1: S_0 B3LYP/def2-TZVP(-f)/CPCM(water) structure of the HBQ-AE molecule: line (left) and ball and stick (right) display style.

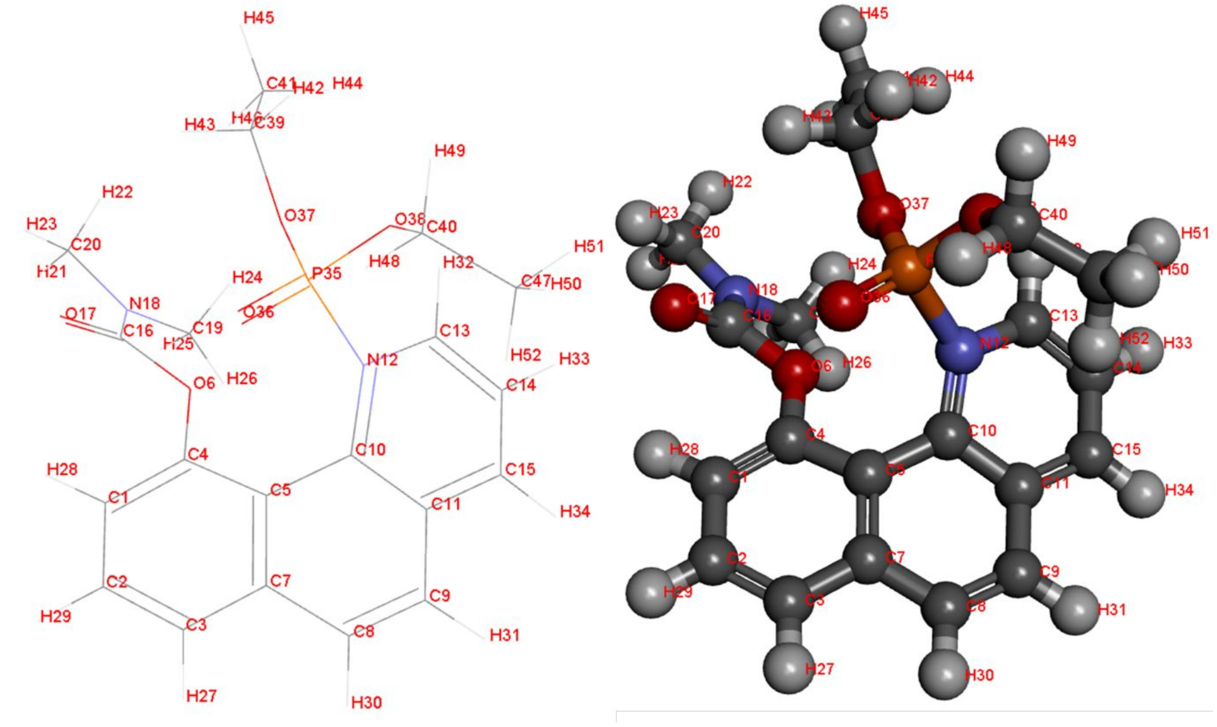

Figure S2: S_0 B3LYP/def2-TZVP(-f)/CPCM(water) structure of the HBQ-DCP molecule: line (left) and ball and stick (right) display style.

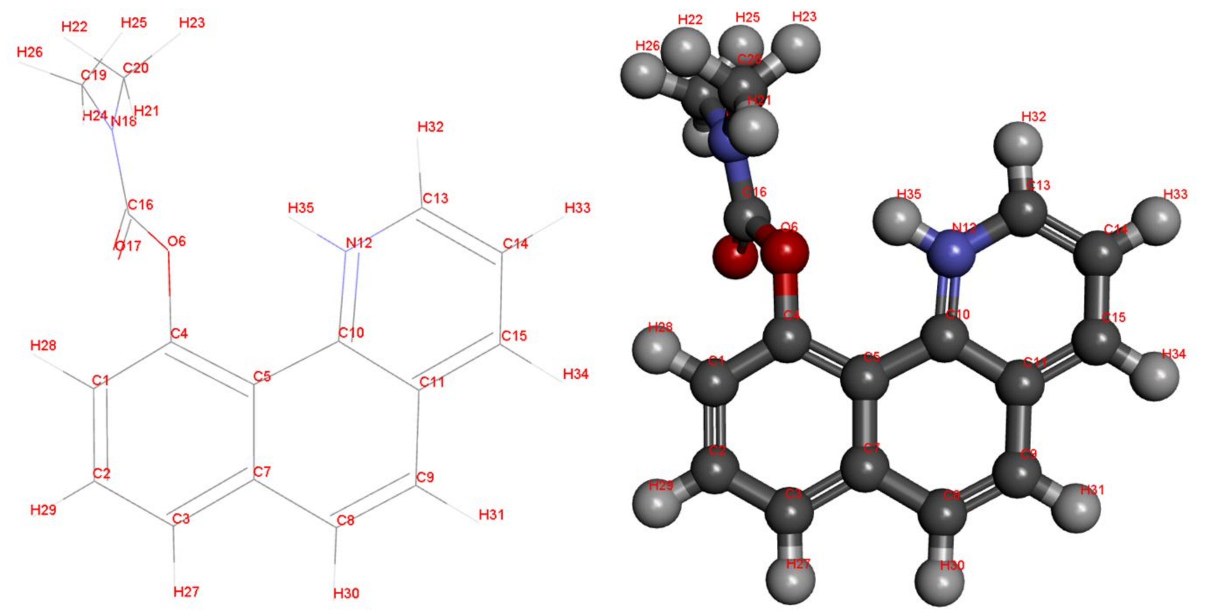

Figure S3: S_0 B3LYP/def2-TZVP(-f)/CPCM(water) structure of the HBQ+H molecule: line (left) and ball and stick (right) display style.

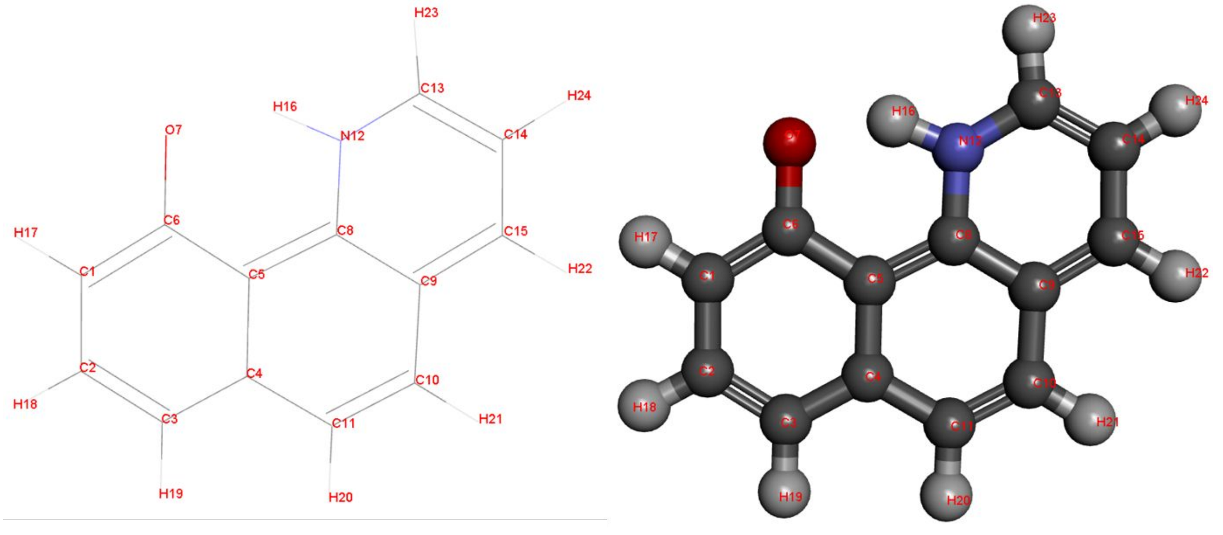

Figure S4: S_0 B3LYP/def2-TZVP(-f)/CPCM(water) structure of the HBQ-Keto molecule: line (left) and ball and stick (right) display style.

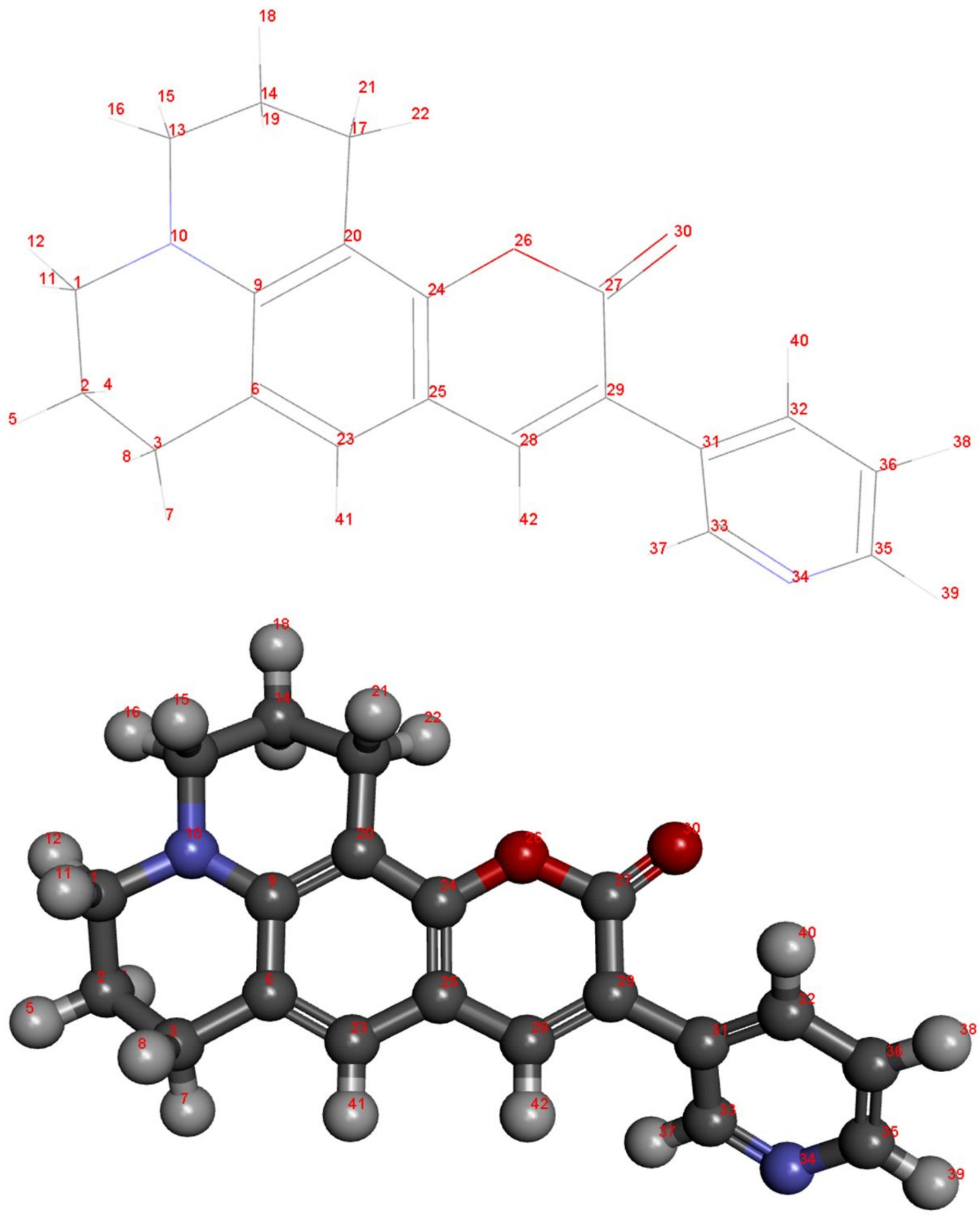

Figure S5: S_0 B3LYP/def2-TZVP(-f)/CPCM(water) structure of the NMU-1 molecule: line (up) and ball and stick (down) display style.

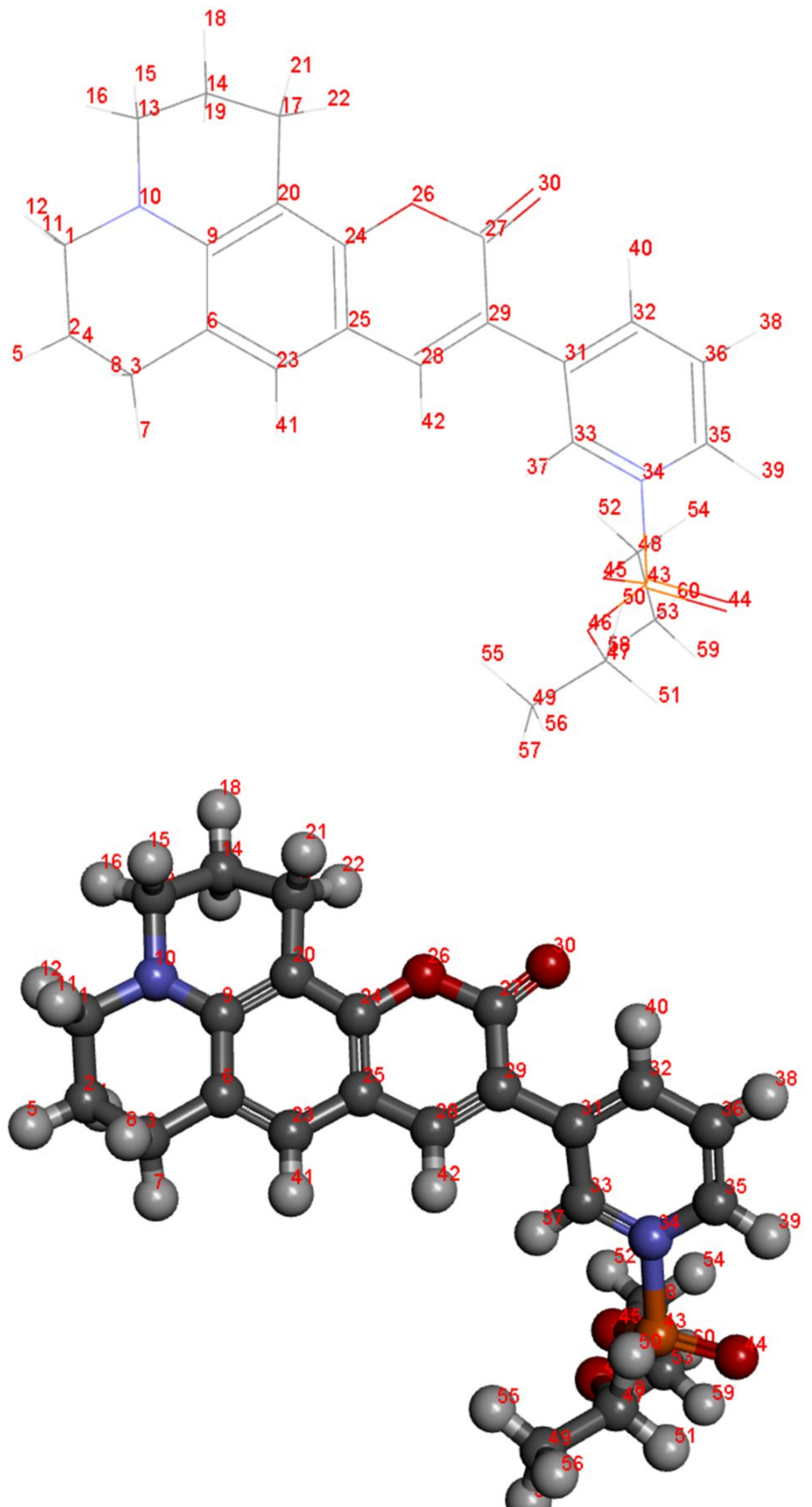

Figure S6: S_0 B3LYP/def2-TZVP(-f)/CPCM(water) structure of the NMU-DCP molecule: line (up) and ball and stick (down) display style.

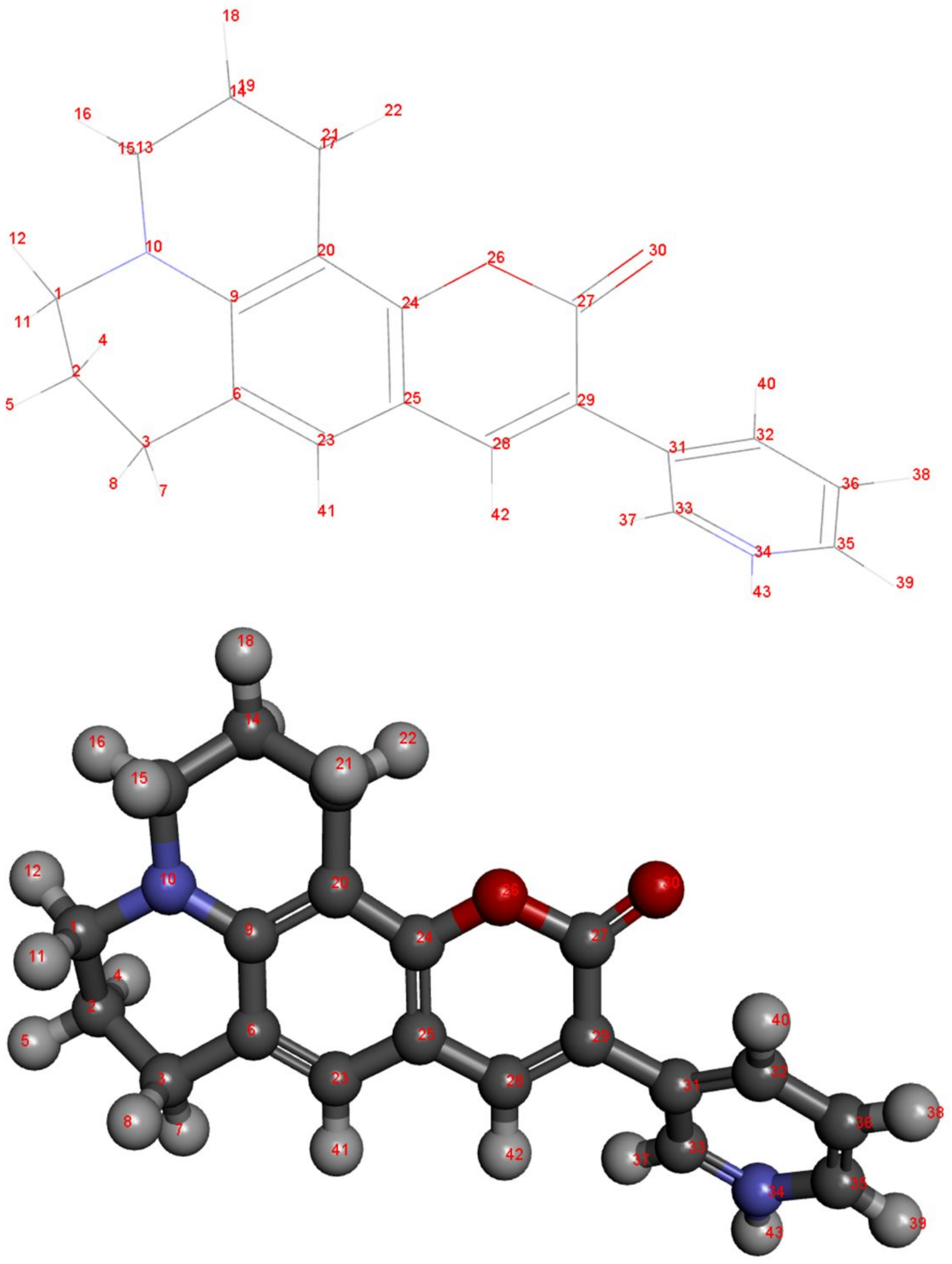

Figure S7: S_0 B3LYP/def2-TZVP(-f)/CPCM(water) structure of the NMU+H molecule: line (up) and ball and stick (down) display style.

Table S2: B3LYP/Def2-TZVP(-f)/CPCM(water) fluorescence energy, oscillator strengths, and charge transfer (CT) decomposition for all molecules. All the q values in units of elementary electric charge (e). Notation: $q_{CT}(AB)$ is the CT from fragment A to fragment B, $q_{CT}(AC)$is the CT from fragment A to fragment C, $q_{CT}(AD)$is the CT from fragment A to fragment D, $q_{CT}(BA)$ is the CT from fragment B to fragment A, q_{CT} (BC) is the CT from fragment B to fragment C, $q_{CT}(BD)$ is the CT from fragment B to fragment D, $q_{CT}(CA)$ is the CT from fragment C to fragment A, $q_{CT}(CB)$ is the CT from fragment C to fragment B, $q_{CT}(CD)$ is the CT from fragment C to fragment D, $q_{CT}(DA)$ is the CT from fragment D to fragment A, $q_{CT}(DB)$ is the CT from fragment D to fragment B, $q_{CT}(DC)$ is the CT from fragment D to fragment C, $q_{LE}(A)$ is the Local Excitation (LE) on fragment A, $q_{LE}(B)$ is the L) on fragment B, $q_{LE}(C)$ is the LE on fragment C, and $q_{LE}(D)$ is the Local Excitation on fragment D.

Molecule	E (eV)	f	$q_{CT}(AB)$	$q_{CT}(AC)$	$q_{CT}(AD)$	$q_{CT}(BA)$	$q_{CT}(BC)$	$q_{CT}(BD)$	$q_{CT}(CA)$	$q_{CT}(CB)$	$q_{CT}(CD)$	$q_{CT}(DA)$	$q_{CT}(DB)$	$q_{CT}(DC)$	$q_{LE}(A)$	$q_{LE}(B)$	$q_{LE}(C)$	$q_{LE}(D)$
HBQ-AE	3.89	0.21	0.004	0.002	–	0.019	0.133	–	0.016	0.356	–	–	–	–	0.001	0.339	0.131	–
HBQ-DCP	2.947	0.148	0.006	0.001	0.000	0.025	0.033	0.002	0.083	0.649	0.005	0.005	0.035	0.006	0.001	0.196	0.102	0.001
HBQ-H	3.355	0.236	0.008	0.002	–	0.017	0.043	–	0.049	0.569	–	–	–	–	0.001	0.201	0.111	–
HBQ-Keto	2.693	0.401	0.008	0.002	–	0.071	0.054	–	0.136	0.506	–	–	–	–	0.002	0.282	0.094	–
NMU-1	3.007	1.266	0.058	0.023	–	0.426	0.028	–	0.096	0.027	–	–	–	–	0.245	0.088	0.010	–
NMU-DCP	2.288	0.084	0.006	0.002	0.000	0.023	0.002	0.000	0.666	0.174	0.001	0.031	0.008	0.003	0.020	0.006	0.058	0.000
NMU-H	2.536	0.253	0.026	0.010	–	0.113	0.010	–	0.540	0.119	–	–	–	–	0.116	0.026	0.041	–

2. Test calculations: dispersion corrections for geometry and range-separated functional for CT decomposition

To validate the chosen theoretical level for geometry optimizations, test calculations were carried out with two representative molecules, NMU-1 and HBQ+H, since both species are directly responsible for the experimentally observed fluorescence.

To assess the influence of dispersion (D3) corrections on the optimized geometries, we compared structures obtained at the B3LYP/def2-TZVP(-f)/CPCM(water) and B3LYP-D3/def2-TZVP(-f)/CPCM(water) levels of theory. The second uses the atom-pairwise dispersion correction with the Becke-Johnson damping scheme (D3BJ). The structural similarity between the two sets of geometries was quantified using the root-mean-square deviation ($RMSD$), defined as:

$$RMSD = \sqrt{\frac{1}{N}\sum_{i=1}^{N}(v_i - w_i)^2}$$

where N is the number of atoms in the molecule, and v_i and w_i denote the Cartesian coordinates of the atoms in the B3LYP and B3LYP-D3 optimized structures, respectively. Therefore, $v_i - w_i$ is the Euclidean distance between the ith pair of corresponding atoms. By employing the $RMSD$ tool within the Discovery Studio visualizer software, the structural superposition (Figure S8) and subsequent $RMSD$ calculation enable a quantitative assessment of the degree of structural similarity between the generated geometries.

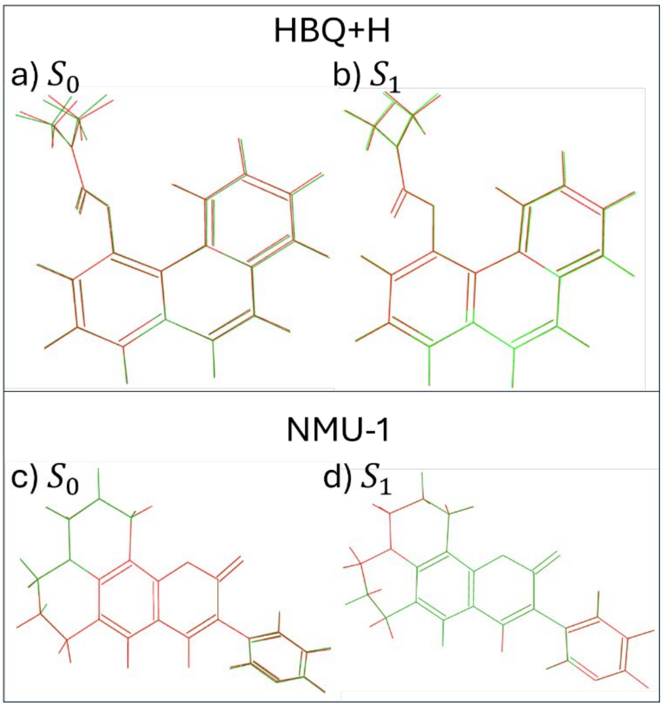

Figure S8. Superimposed geometries of the HBQ+H (a) S_0 and (b) S_1 states, and NMU-1 (c) S_0 and (d) S_1 states. The B3LYP/def2-TZVP(-f) geometries are green colored, and the B3LYP-D3/def2-TZVP(-f) geometries are red colored.

The resulting $RMSD$ values for the ground state (S_0) geometries were 0.1064 Å for HBQ+H and 0.0340 Å for NMU-1, indicating that the geometries are virtually identical. For the first excited singlet state (S_1), the deviations were even smaller (0.0436 Å for HBQ+H and 0.0150 Å for NMU-1). Superimposed structures are shown in Figure SX. These results demonstrate that the inclusion of dispersion corrections has a small effect on the optimized geometries of the systems investigated, validating the use of the chosen theoretical level.

To further evaluate the description of the CT character, we additionally employed the CAM-B3LYP functional, which belongs to the class of range-separated hybrids known to improve the description of CT excitations.[1–3] Again, HBQ+H and NMU-1 were selected as representative systems. The CT decomposition analysis was performed at both B3LYP/def2-TZVP(-f)/CPCM(water) and CAM-B3LYP/def2-TZVP(-f)/CPCM(water) levels.

Tabel S3: B3LYP and CAM-B3LYP functionals with def2-TZVP(-f)/CPCM(water) charge transfer (CT) decomposition for HBQ+H and NMU-1 molecules. All the q values in units of elementary electric charge (e). Notation: $q_{CT}(AB)$ is the CT from fragment A to fragment B, $q_{CT}(AC)$is the CT from fragment A to fragment C, $q_{CT}(BA)$ is the CT from fragment B to fragment A, $q_{CT}(BC)$ is the CT from fragment B to fragment C, q_{CT} (CA) is the CT from fragment C to fragment A, $q_{CT}(CB)$ is the CT from fragment C to fragment B, $q_{LE}(A)$ is the Local Excitation (LE) on fragment A, $q_{LE}(B)$ is the L) on fragment B, and $q_{LE}(C)$ is the LE on fragment C.

HBQ+H									
**Functional**	**CTAB**	**CTAC**	**CTBA**	**CTCA**	**CTBC**	**CTCB**	**LEA**	**LEB**	**LEC**
B3LYP	0.008	0.002	0.017	0.049	0.043	0.569	0.001	0.201	0.111
CAM-B3LYP	0.005	0.002	0.045	0.052	0.062	0.453	0.002	0.234	0.145
Error (%)	63.0%	18.1%	63.0%	5.4%	31.4%	25.6%	52.7%	14.2%	23.5%
**NMU-1**									
**Functional**	**CTAB**	**CTAC**	**CTBA**	**CTCA**	**CTBC**	**CTCB**	**LEA**	**LEB**	**LEC**
B3LYP	0.058	0.023	0.426	0.096	0.028	0.027	0.245	0.088	0.010
CAM-B3LYP	0.057	0.026	0.392	0.134	0.018	0.023	0.281	0.056	0.013
Error (%)	0.3%	11.5%	8.9%	28.5%	58.4%	15.9%	13.0%	58.0%	26.3%

The results are summarized in Table S3. Although the absolute contributions differ between B3LYP and CAM-B3LYP, the main trends remain unchanged. For HBQ+H, the dominant contribution is consistently identified as $q_{CT}(CB)$, with the second largest being a Local Excitation (LE) on fragment B, $q_{LE}(B)$. Similarly, for NMU-1, the main contribution is $q_{CT}(BA)$ on both levels of theory, followed by $q_{LE}(A)$. Overall, while B3LYP slightly overestimates some CT components (e.g., by 25.6% for $q_{CT}(CB)$ of HBQ+H and 8.9% for $q_{CT}(BA)$ of NMU-1), the overall patterns and conclusions are the same.

These test calculations confirm that the theoretical level employed in the present study is consistent. The inclusion of DFT-D corrections leads to small differences in optimized geometries, and the comparison with CAM-B3LYP shows that the relative pattern of CT contributions is maintained. Therefore, the main conclusions of the manuscript remain fully supported.

3. References

(1) Cai, Z.-L.; Crossley, M. J.; Reimers, J. R.; Kobayashi, R.; Amos, R. D. Density Functional Theory for Charge Transfer: The Nature of the N-Bands of Porphyrins and Chlorophylls Revealed through CAM-B3LYP, CASPT2, and SAC-CI Calculations. *J Phys Chem B* **2006**, *110* (31), 15624–15632. https://doi.org/10.1021/jp063376t.

(2) Máximo-Canadas, M.; Borges, I. Absorption Spectra of P-Nitroaniline Derivatives: Charge Transfer Effects and the Role of Substituents. *J Mol Model* **2024**, *30* (5), 120. https://doi.org/10.1007/s00894-024-05917-0.

(3) Máximo-Canadas, M.; Modesto-Costa, L.; Borges, I. Ab Initio Electronic Absorption Spectra of Para-nitroaniline in Different Solvents: Intramolecular Charge Transfer Effects. *J Comput Chem* **2024**. https://doi.org/10.1002/jcc.27493.
